# Exogenous γ-aminobutyric acid mitigates physiological and transcriptomic responses of *Prunus mongolica* under combined alkali salt and cadmium stress

**DOI:** 10.3389/fpls.2026.1927917

**Published:** 2026-08-25

**Authors:** Guiquan Wang, Lei Wang, Xuebo Li, Lu Cao, Youwei Zhang, Heyi Wang, Xiaomin Zhang, Ze Ren, Yunpeng Zhang, Yu Wang

**Affiliations:** 1 Forestry College, Inner Mongolia Agricultural University, Hohhot, China; 2 Architecture College, Tianjin University, Tianjin, China; 3 Grasslands College, Inner Mongolia Agricultural University, Hohhot, China; 4 Inner Mongolia Expressway Shanmei Ecological Development Co., Ltd., Hohhot, China

**Keywords:** combined alkali salt and cadmium stress, gene expression response, metabolic reprogramming, *Prunus mongolica*, transcriptome analysis, γ-aminobutyric acid

## Abstract

**Introduction:**

*Prunus mongolica* is a relict xerophytic shrub important for desert ecosystem stability and vegetation restoration in arid regions. However, its adaptive mechanisms under combined alkali salt and cadmium stress remain unclear. This study investigated the regulatory role of exogenous γ-aminobutyric acid (GABA) in stress tolerance under single and combined stresses.

**Methods:**

In this study, seedlings were subjected to alkali salt, cadmium, and combined stress treatments, followed by foliar application of GABA. Germination, growth, physiological traits, and transcriptomic responses were analyzed using an integrated multi-level approach.

**Results:**

The alkali salt, cadmium, and combined alkali salt and cadmium stress significantly inhibited germination and growth, with combined stress showing the strongest effects. Stress induced reactive oxygen species accumulation, lipid peroxidation, and photosynthetic inhibition. Exogenous γ-aminobutyric acid alleviated these effects by improving germination, growth, chlorophyll content, and root activity, while reducing oxidative damage. It enhanced osmotic adjustment via increased proline and soluble sugars and strengthened antioxidant enzyme activities, including superoxide dismutase, peroxidase, catalase, ascorbate peroxidase, and glutathione reductase, restoring redox homeostasis.

**Discussion:**

Transcriptome analysis showed that GABA reprogrammed stress-responsive genes enriched in “MAPK signaling pathway–plant”, “Plant hormone signal transduction”, “Plant–pathogen interaction”, “Peroxisome”, and “Glutathione metabolism” pathways. Gene set enrichment analysis further indicated recovery of photosynthesis, carbon metabolism, and primary “metabolic process”, suggesting restoration of energy balance and metabolic homeostasis. Collectively, GABA functions as a multi-level regulator integrating redox balance, signaling pathways, and metabolic reprogramming to enhance stress tolerance in *Prunus mongolica*.

## 1 Introduction

Soil alkalization and salinization are major environmental constraints affecting global agricultural productivity and ecosystem stability, particularly in arid and semi-arid regions, where they severely limit agricultural production, ecological restoration, and vegetation recovery (Shrivastava and Kumar, 2015; Yang et al., 2022). Saline–alkali soils are typically characterized by high salt concentrations, elevated pH, and accumulation of exchangeable sodium, accompanied by soil structural degradation and reduced nutrient availability, thereby severely restricting plant growth and ecosystem restoration capacity (Munns and Tester, 2008; Zhu, 2016; Yang and Guo, 2018; Zelm et al., 2020). Alkali salt stress not only induces ionic toxicity and osmotic stress but also exacerbates mineral nutrient imbalance due to high pH conditions, thereby imposing a significantly stronger combined stress on plants than neutral salt stress (Wang et al., 2022; Liu et al., 2022; Feng et al., 2024). At the plant response level, both alkali salt stress and heavy metal stress can induce excessive accumulation of reactive oxygen species (ROS), disrupt membrane system stability, and inhibit photosynthetic processes, ultimately leading to growth inhibition or even plant death (Foyer and Noctor, 2011; Hasanuzzaman et al., 2020). Cd, as a typical heavy metal, can further disrupt ion homeostasis and the balance of antioxidant systems, thereby exacerbating photosynthetic inhibition and metabolic disorders (An et al., 2021; Zulfiqar et al., 2022). Therefore, under combined stress conditions, plants generally exhibit more severe oxidative damage and systemic metabolic imbalance (Sánchez-Bermúdez et al., 2022; Cui et al., 2022; Yue et al., 2024).

*Prunus mongolica* is a deciduous shrub of the genus *Prunus* (Rosaceae) and an ancient relict species endemic to the Mongolian Plateau and the Gobi Desert of Central Asia (Ma et al., 2014; Liu et al., 2025). This species is a typical component of the Alashan Desert region and is mainly distributed across the desert–grassland ecotones of the Alashan Gobi in Inner Mongolia, the Hexi Corridor in Gansu Province, the Helan Mountains in Ningxia, and the Altay region in Xinjiang, China (Si and Xiu, 2007; Feng et al., 2025). Due to long-term human activities such as fruit harvesting, fuelwood collection, and overgrazing, as well as its reproductive characteristics as an outcrossing species dependent on insect pollination with limited pollen dispersal distance, this species exhibits low seed set and a significant decline in population size (Fang et al., 2007). Accordingly, *Prunus mongolica* has been listed as an endangered species in the *Red Data Book of Chinese Plants* and designated as a second-class nationally protected wild plant species in China (Fu et al., 1991; National Environmental Protection Agency, and Institute of Botany, Chinese Academy of Sciences, 1978).

γ-Aminobutyric acid (GABA) is a widely distributed non-protein amino acid that has been reported to participate in the regulation of carbon and nitrogen metabolic balance as well as plant stress responses (Michaeli and Fromm, 2015; Ramesh and Tyerman, 2015; Bown and Shelp, 2016). Accumulating evidence has shown that GABA has emerged as a signaling hub linking stress perception and metabolic reprogramming (Ramos-Ruiz et al., 2019; Li et al., 2020; Hasan et al., 2021; Dabravolski and Isayenkov, 2023; Yuan et al., 2023), and it can enhance plant tolerance to salt and drought stresses by regulating the AsA–GSH cycle, improving antioxidant system activity, and maintaining ion homeostasis (Jia et al., 2021; Ullah et al., 2023; Zhao et al., 2024). In addition, GABA is also involved in signal transduction and metabolic network reprogramming, thereby coordinating the balance between plant growth and “defense response” (Li Y. X. et al., 2022; Zhang et al., 2025; Dai et al., 2025).

However, current studies have mainly focused on the regulatory roles of GABA under single salt or drought stress conditions. Its systemic regulatory mechanisms under combined alkali salt and heavy metal stress remain insufficiently understood, particularly the integrative relationships among physiological responses, transcriptional regulation, and metabolic network reprogramming. Therefore, using *Prunus mongolica* as the experimental material, this study systematically evaluated the effects of exogenous GABA on seed germination, seedling growth, and physiological traits under alkali salt, cadmium, and combined stress conditions. Furthermore, transcriptomic and pathway enrichment analyses were conducted to elucidate the underlying molecular regulatory mechanisms, aiming to provide theoretical insights into plant stress tolerance mechanisms and ecological restoration in arid regions.

## 2 Materials and methods

The overall study was carried out from August 2025 to February 2026 in the Landscape Plant and Ornamental Horticulture Laboratory of the College of Forestry, Inner Mongolia Agricultural University, as well as in a controlled intelligent greenhouse (111°41′E–111°37′E, 40°48′N–40°68′N). This period included seed collection, dry storage, preliminary concentration screening, the 21-day germination and seedling treatment experiment, and subsequent physiological and transcriptomic analyses. The seeds of *Prunus mongolica* used in this study were collected from wild populations in Wuhai City, Inner Mongolia Autonomous Region, China (106°48′E–109°31′E, 38°45′N–40°45′N) in August 2025. After natural air-drying, the seeds were dry-stored at 4 °C until use to maintain seed viability and material consistency. No moist cold stratification treatment was applied for dormancy breaking before the experiment.

### 2.1 Experimental layout and design

After dry storage at 4 °C, seeds were removed and allowed to equilibrate to room temperature before the experiment. Healthy and uniformly sized seeds were then selected and surface-sterilized in 0.05% KMnO_4_ for 25 min, followed by rinsing with sterile distilled water six times. Based on preliminary experiments, seeds were pre-soaked in distilled water for 12 h, and sinking seeds were selected for subsequent germination experiments.

Based on previous studies conducted by our research group on the physicochemical properties of different soil types in Wuhai City, Inner Mongolia Autonomous Region, China (**Supplementary Table 1**), the local soil pH was mainly distributed in the range of 7.50–8.60. To better reflect the ecological restoration conditions in this region, the pH range for simulated alkali salt stress was adjusted to 8.50–8.80 in this experiment. Preliminary experiments conducted by our research group showed that treatment with a 10.00 mM mixed alkali salt solution (prepared with 0.81 g/L NaHCO_3_ and 0.036 g/L Na_2_CO_3_, analytical grade) and 0.50 mM cadmium solution (prepared from 97.00 mg/L CdCl_2_·H_2_O, analytical grade) significantly inhibited seed germination and seedling growth of *Prunus mongolica*. In contrast, 0.90 mM exogenous GABA treatment (prepared using 92.8 mg/L γ-aminobutyric acid, analytical grade) significantly promoted seed germination and seedling development. Therefore, these concentrations were selected to simulate alkali salt stress, cadmium stress, combined alkali salt–cadmium stress, and exogenous GABA treatment in this study.

The experimental substrate consisted of a fertilized white peat-based seedling medium (main components: 30% *Sphagnum* peat and white peat; 70% black peat derived from *Sphagnum* peat), purchased from Floragard GmbH, Germany. The initial physicochemical properties of the substrate are provided in **Supplementary Table 2**. Prior to use, the substrate was thoroughly mixed with perlite at a volume ratio of 3:1 (substrate:perlite). It was then saturated with 0.3% KMnO_4_ solution (analytical grade) for disinfection, sealed with polyethylene film for 12 h, and subsequently exposed to sunlight for 8 h (daytime temperature 28–32 °C, UV intensity ≥ 1800 μW/cm²) to inactivate potential pathogens and degrade residual disinfectant. The experimental containers were black polypropylene (PP) seedling trays with 32 cells. Each cell had an upper diameter of 60 mm, lower diameter of 26 mm, and depth of 53 mm, with a volume of approximately 110 mL. The overall tray dimensions were 54 cm (length) × 28 cm (width) × 5.5 cm (height). Prior to use, the trays were soaked in 75% ethanol for 20 min, rinsed three times with distilled water, and air-dried under sterile ventilation conditions. One seed was sown in each cell. Three 32-cell seedling trays were grouped as one experimental unit, namely one biological replicate, containing 96 seeds.

A total of eight treatment groups were established in this experiment: CK (distilled water control), G (GABA), S (alkali salt stress), Cd (cadmium stress), SCd (combined alkali salt and cadmium stress), SG (alkali salt stress + GABA), CdG (cadmium stress + GABA), and SCdG (combined alkali salt and cadmium stress + GABA). During the germination stage, substrate moisture was maintained by adding the corresponding treatment solutions to each treatment group. After seedling emergence, foliar spraying was conducted daily at 18:00 until the end of the 21-day experiment. For GABA-containing treatments, 0.90 mM GABA solution was applied by foliar spraying, whereas the non-GABA treatments received an equal volume of distilled water. The corresponding alkali salt and/or Cd stress solutions were continuously supplied through substrate irrigation to maintain the stress conditions throughout the experiment. The growth conditions were maintained at a day/night temperature of 25 ± 1 °C/18 ± 1 °C, relative humidity of 50–60%, a 12 h light/12 h dark photoperiod, and a light intensity of 5500 lx. The 21-day experimental period referred to the germination and seedling treatment stage, starting from sowing and ending at the final sampling point. Each treatment included three independent biological replicates. Seed germination was recorded daily, and the experiment was considered complete when no new germination was observed for seven consecutive days. At the end of the 21-day treatment period, seedlings with similar growth status were selected from each experimental unit for physiological measurements and transcriptome sequencing. Physiological and transcriptomic samples were collected at the same sampling time point. For RNA-seq, fresh leaf tissues were collected from upper fully expanded leaves of seedlings with similar growth status and developmental stage. Because leaf number differed among treatments at the early seedling stage, RNA-seq sampling was standardized by leaf developmental status and relative position rather than by a fixed numerical node. For physiological and biochemical measurements, leaf and root tissues were collected separately according to the requirements of each indicator, rather than using whole seedlings as mixed samples.

### 2.2 Experimental methods

#### 2.2.1 Seed germination and seedling survival indicators

The first day after sowing, namely 24 h after sowing, was defined as day 1 of the germination experiment. Thereafter, germinated seeds in each experimental unit were counted every 24 h until the termination of germination. The germination experiment was terminated at the end of the 21-day experimental period, when no new germination had been observed for seven consecutive days. At this time, the death rate (Equation 1), germination potential (Equation 2), germination rate (Equation 3), germination index (Equation 4), and vigor index (Equation 5) were calculated. The seed germination test was conducted following the International Seed Testing Association (ISTA) standards (International Seed Testing Association, 2023). Germination potential was defined as the time point when the number of germinated seeds reached its maximum peak. The calculations of death rate (DR), germination potential (GP), and germination rate followed the ISTA standard, while the germination index (GI) and vigor index (VI) were calculated according to classical methods in seed vigor research (Coolbear et al., 1984; Bewley et al., 2013). The specific calculation formulas are as follows:

(1) $$DR\;(\%)\;=\frac{Number\;of\;dead\;seedlings}{N}\times 100$$

(2) $$GP\;(\%)=\frac{n_{0}}{N}\times 100$$

(3) $$Germination\;rate\;(\%)=\frac{n}{N}\times 100$$

(4) $$GI=\sum (n_{i}\times d_{i})$$

(5) $$VI=GI\times (L_{1}\times L_{2})$$

Where DR(%) represents the death rate; GP(%) represents germination potential; GI represents germination index; and VI represents vigor index; n represents the number of normally germinated seeds during the 21-day experimental period, and N represents the total number of seeds in each experimental unit. d_i_ represents the number of days from sowing, and n_i_ represents the number of normally germinated seeds on day i; L_1_ represents stem length (SL), and L_2_ represents root length (RL). For germination-related indices, values were calculated separately for each biological replicate and then averaged across three biological replicates for each treatment.

#### 2.2.2 Seedling morphological traits

For morphological measurements, three seedlings were randomly selected from each biological replicate, resulting in nine seedlings per treatment group. Seedling surface soil was gently removed by rinsing with distilled water, and surface moisture was blotted dry using filter paper. The seedlings were then placed flat on a clean surface for morphological measurements. The number of leaves (LN) and lateral roots (LRN) was recorded for each selected seedling, and LN was expressed as the mean leaf number per seedling for each biological replicate. Seedling height (SH) referred to the vertical distance from the root–shoot junction to the highest point of the seedling, whereas stem length (SL) referred to the length of the main stem measured along the stem axis from the root–shoot junction to the shoot apex. Root length (RL) was measured from the root–shoot junction to the root tip. Stem diameter (SD) was measured at the basal stem near the root–shoot junction, close to the substrate surface. All length and diameter measurements were performed using a digital vernier caliper with a precision of 0.001 mm. To minimize variation caused by differences in leaf age and developmental stage, leaf length (LL) and leaf width (LW) were measured using fully expanded leaves at a fixed top position, following Cui et al. (2025) with slight modifications. Specifically, the uppermost fully expanded leaf and the next two consecutive fully expanded leaves below it were selected from each seedling, and their mean values were used as the final LL and LW for each seedling.

#### 2.2.3 Physiological parameters

For physiological and biochemical measurements, healthy seedlings with similar growth performance were randomly selected from each experimental unit, namely each biological replicate. Physiological and biochemical measurements were not performed using whole seedlings as mixed samples. Instead, different tissues were collected separately according to the requirements of each indicator. Fresh leaf tissues were used to determine photosynthetic pigments, oxidative damage indicators, osmotic adjustment substances, antioxidant enzyme activities, AsA, GSH, and RWC. Root tissues were collected separately and used only for root activity measurement using the TTC method. These tissues were harvested and immediately transported to the Landscape Plant Laboratory of the College of Forestry, Inner Mongolia Agricultural University for physiological measurements. Based on the method of Zou (2000), superoxide dismutase (SOD) activity was determined using the nitro blue tetrazolium (NBT) method, peroxidase (POD) activity was measured using the guaiacol colorimetric method, catalase (CAT) activity was determined by ultraviolet absorption spectrophotometry, and ascorbate peroxidase (APX) activity was measured using spectrophotometric methods. According to Zhang et al. (2009), malondialdehyde (MDA) content was determined using the thiobarbituric acid (TBA) method. Following Li (2000), soluble sugar (SS) content was measured using the anthrone colorimetric method, soluble protein (SP) content was determined by ultraviolet spectrophotometry, chlorophyll content was measured by the direct extraction method, and root activity was determined using the TTC method. Hydrogen peroxide (H_2_O_2_) and superoxide anion (O_2_^-^) contents were determined following Shi (2016). Ascorbic acid (AsA) content was measured using spectrophotometry, and free proline (Pro) was extracted using sulfosalicylic acid according to Gao and Cai (2018). Relative water content (RWC) of plant tissues was determined according to Gao and Cai (2018) and Wang and Huang (2015). Polyphenol oxidase (PPO) activity, reduced glutathione (GSH) content, and glutathione reductase (GR) activity were determined using commercial assay kits following the manufacturer’s instructions. All measurements were performed with three biological replicates, and the mean values were calculated.

#### 2.2.4 RNA extraction and transcriptome sequencing

For each treatment, three independent biological replicates were prepared. For each experimental unit, namely each biological replicate, 0.2 g of fresh leaf tissue was collected from fully expanded leaves of seedlings with similar growth status. Leaf tissues were pooled only within the same experimental unit and used as one RNA sequencing (RNA-seq) sample. Total RNA was isolated and purified using TRIzol reagent (RNA extraction kit). The quality and purity of RNA were assessed, and RNA integrity was evaluated using an Agilent 2100 Bioanalyzer. Only high-quality RNA samples (concentration > 50 ng/μL, RNA integrity number (RIN) > 7.0, and total RNA > 1 μg) were used for subsequent analyses. Qualified RNA samples were used for library construction. mRNA was enriched using oligo(dT) magnetic beads to capture poly(A)-tailed transcripts. The purified mRNA was then subjected to fragmentation, first-strand cDNA synthesis, second-strand cDNA synthesis, end repair, A-tailing, adapter ligation, fragment size selection, and PCR amplification to construct strand-specific transcriptome libraries. The insert size of the libraries was approximately 300 bp ± 50 bp. After quality control, the libraries were sequenced on an Illumina NovaSeq™ X Plus platform using a paired-end 150 bp (PE150) sequencing strategy. Sequencing was performed by LC-Bio Technology Co., Ltd. (Hangzhou, China).

#### 2.2.5 Transcriptome functional annotation

Transcriptome sequences were functionally annotated by aligning against multiple public databases. BLAST software was used to compare transcript sequences against the NCBI non-redundant protein (NR), Swiss-Prot, Gene Ontology (GO), Clusters of Orthologous Groups (COG), Eukaryotic Orthologous Groups (KOG), and Kyoto Encyclopedia of Genes and Genomes (KEGG) databases to obtain preliminary functional annotations. The amino acid sequences of transcripts were predicted prior to further analysis. Subsequently, HMMER software was used to search against the Pfam protein family database to identify conserved domains and obtain comprehensive functional annotation information.

#### 2.2.6 qRT-PCR validation

To validate the RNA-seq results and analyze the regulatory effects of exogenous GABA on stress-responsive gene expression in *Prunus mongolica*, five stress-related genes were selected for quantitative real-time PCR (qRT-PCR) analysis, using *β-actin* as the internal reference gene. Primer sequences used in this study are listed in **Supplementary Table 3**. The PCR amplification program was as follows: initial denaturation at 95 °C for 30 s, followed by 40 cycles of 95 °C for 5 s and 60 °C for 20 s. A melting curve analysis was subsequently performed, with temperature increasing from 60 °C to 95 °C, and fluorescence signals collected at each 1 °C increment. All reactions were performed with three biological replicates. Relative gene expression levels were calculated using the 2^-ΔΔCt^ method.

#### 2.2.7 Data processing and analysis

Differentially expressed genes (DEGs) were identified using DESeq2 software, with thresholds set as |log_2_FC| ≥ 1 and false discovery rate (FDR) < 0.05. Fold change represents the ratio of gene expression levels between two sample groups, while *P*-value indicates the statistical significance of differential expression. Plant transcription factor prediction was performed using iTAK software (v1.7a). To further investigate the involvement of DEGs in biological metabolic process, comprehensive pathway annotation and enrichment analyses were performed based on the DEG list. The enrichment factor indicates the degree of enrichment, with higher values representing stronger enrichment. The q-value is the FDR-adjusted P-value obtained after multiple hypothesis testing correction, ranging from 0 to 1, where values closer to zero indicate higher statistical significance.

All germination, seedling morphological, and physiological and biochemical data were analyzed using SPSS Statistics 25 software. One-way analysis of variance (ANOVA) was performed to evaluate differences among treatments. All data are presented as mean ± standard deviation (mean ± SD), calculated from three biological replicates per treatment. Statistical significance among treatments was determined using the least significant difference (LSD) test at *P* < 0.05. Figures and tables were generated using Microsoft Excel 2019 and Origin 2022. Correlation analysis between physiological parameters and transcriptomic data was performed using the Chiplot platform (https://www.chiplot.online/) and the OmicStudio cloud platform (https://www.omicstudio.cn/home?slide=3) for bioinformatics analysis and visualization.

Relative percentage changes reported in the Results section were calculated using the corresponding reference treatment as the denominator. Stress-induced changes and the effect of GABA alone were calculated relative to CK, whereas GABA-mediated alleviating effects under stress conditions were calculated by comparing each GABA-treated stress group with its corresponding stress treatment, namely SG vs. S, CdG vs. Cd, and SCdG vs. SCd. Positive values indicated increases relative to the reference treatment, whereas negative values indicated decreases. For growth- or germination-related positive indicators, increases were interpreted as promotion or recovery, whereas decreases were interpreted as inhibition. For stress-damage indicators, such as DR, MDA, H_2_O_2_, and O_2_^-^, decreases were interpreted as alleviation of stress-induced damage.

## 3 Results

### 3.1 Effects of exogenous GABA on seed germination and seedling morphology

As shown in **Table 1**, exogenous GABA significantly promoted seed germination of *Prunus mongolica* compared with the CK (*P* < 0.05). Relative to CK, germination rate, VI, and GI in the G treatment increased by 19.74%, 35.07%, and 8.28%, respectively. The experimental results showed that all stress treatments significantly inhibited seed germination (*P* < 0.05). Among them, SCd showed the strongest inhibitory effects, with germination rate, VI, GI, and GP decreasing by 52.63%, 67.51%, 48.08%, and 56.14%, respectively, while DR increased by 123.08% compared with CK. Exogenous GABA application alleviated the inhibitory effects induced by stress treatments. For example, compared with S, the SG treatment significantly improved all measured indices by 20.97%–90.80%, with germination rate recovering to levels close to CK, and DR decreasing by 20.83%. Compared with Cd treatment, CdG treatment significantly improved all indices, with increases ranging from 17.14%–45.14%. Notably, DR (decreased by 16.67%) and GP were restored to levels close to CK, and germination rate exceeded that of CK. Compared with SCd, SCdG treatment increased all indices by 22.22%–72.22%, and DR decreased by 41.38%. Several indices recovered to or even exceeded CK levels.

**Table 1:** Seed germination characteristics of *Prunus mongolica* under different treatments.

Treatment	Death rate (%)	Germination rate (%)	Germination potential (%)	Germination index	Vigor index (×10²)
CK	14.33± 2.08^d^	75.67 ± 2.08^c^	56.67 ± 4.51^a^	11.96 ± 0.65^b^	23.38 ± 1.52^b^
G	4.67 ± 1.53^e^	91.33 ± 0.58^a^	58.00 ± 3.61^a^	12.92 ± 0.29^a^	31.58 ± 2.37^a^
S	24.67 ± 3.06^b^	61.67 ± 3.51^d^	29.00 ± 2.65^ef^	5.72 ± 0.47^e^	8.94 ± 0.95^e^
Cd	19.67 ± 2.52^c^	70.33 ± 1.53^c^	41.33 ± 2.52^cd^	8.06 ± 0.4^9d^	12.17 ± 1.01^d^
SCd	33.67 ± 4.51^a^	36.33 ± 5.13^e^	25.33 ± 5.03^f^	6.21 ± 0.38^e^	7.60 ± 0.95^e^
SG	18.33 ± 2.31^cd^	75.00 ± 3.00^c^	45.33 ± 5.86^bc^	8.96 ± 0.57^c^	17.06± 0.72^c^
CdG	14.33 ± 0.58^d^	82.33 ± 2.52^b^	51.00 ± 5.57^ab^	9.73 ± 0.37^c^	17.66± 1.21^c^
SCdG	17.67 ± 3.06^cd^	62.33 ± 4.51^d^	34.67 ± 1.53d^e^	7.59 ± 0.60^d^	11.62± 1.07^b^

Values are expressed as mean ± standard deviation (n = 3). Different superscript lowercase letters within a column indicate statistically significant differences among treatments (*P* < 0.05), determined by one-way ANOVA followed by LSD test. The VI was scaled by a factor of 10².

**Figure 1:**
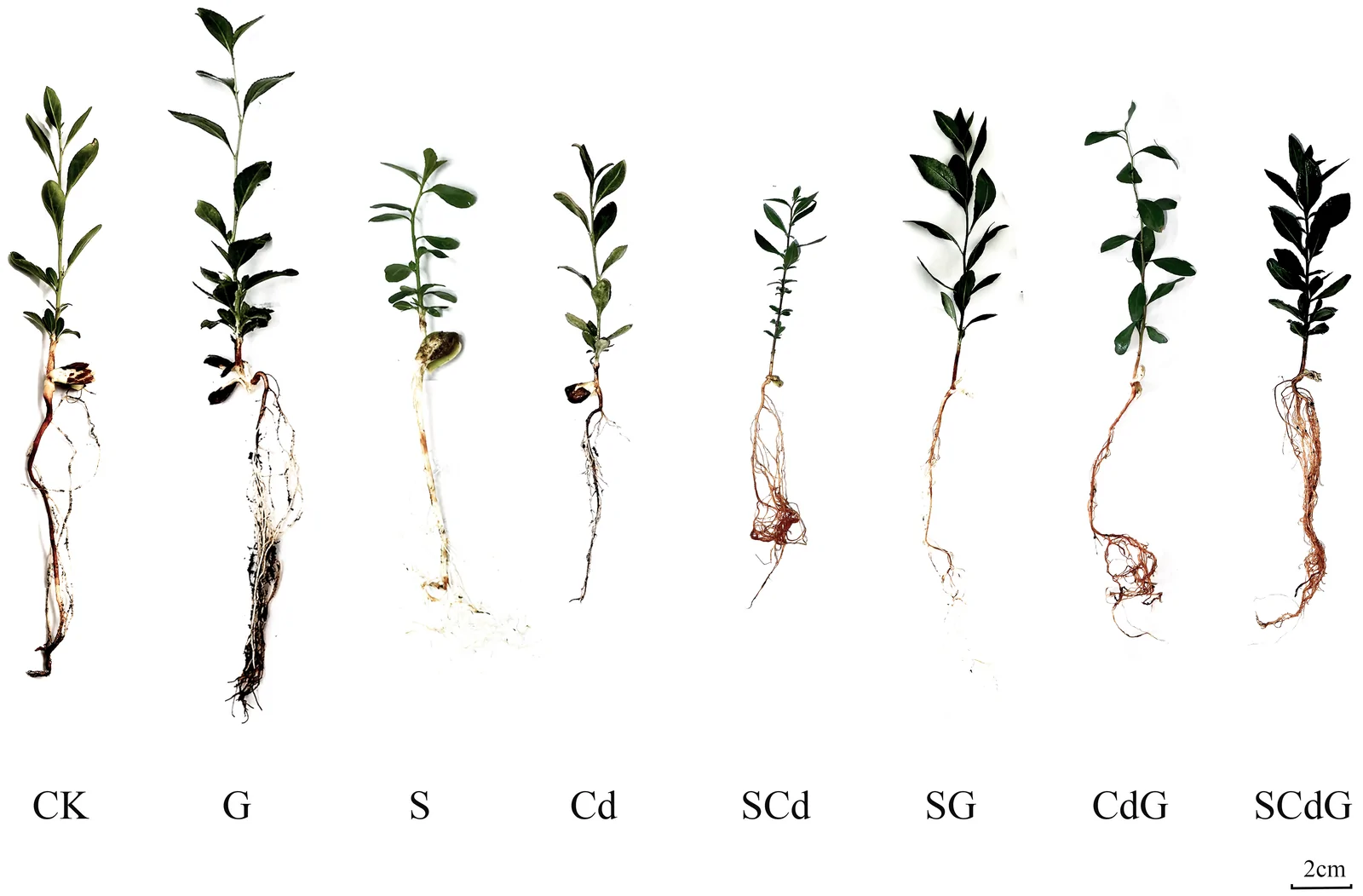
Seedling morphology of *Prunus mongolica* under different treatments. The seedlings were photographed after substrate removal and gentle washing at 21 days after sowing. The images are intended to visually show representative seedling morphology under different treatments.

As shown in **Figure 1**, exogenous GABA effectively alleviated the inhibitory effects of S, Cd, and SCd on seedling growth. It promoted SH, SL, root elongation, and leaf expansion. Under stress conditions, leaf morphology was significantly altered, characterized by narrow and elongated leaves, whereas GABA treatment restored leaf morphology to a relatively broader phenotype. Meanwhile, the results suggest that exogenous GABA may regulate excessive lateral root proliferation under stress conditions, thereby contributing to the optimization of root system architecture.

**Figure 2:**
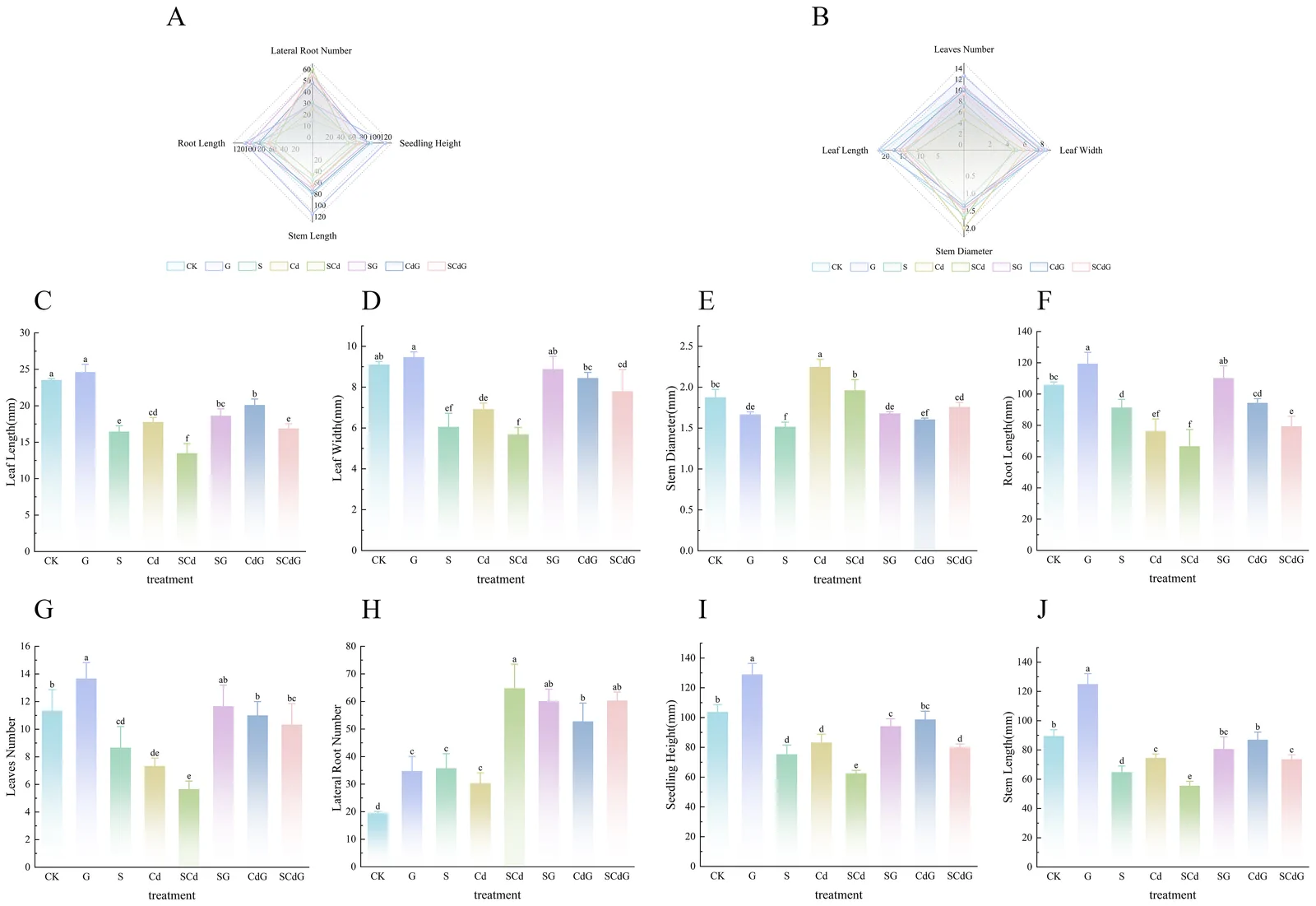
Morphological responses of *Prunus mongolica* seedlings under different treatments. **(A)** is a radar plot of SH, SL, LRN, and RL; **(B)** is a radar plot of LL, LW, LN, and SD; **(C)** is leaf length; **(D)** is leaf width; **(E)** is stem diameter; **(F)** is root length; **(G)** is leaf number per seedling; **(H)** is lateral root number; **(I)** is seedling height; **(J)** is stem length. Different colors indicate different treatments, and different lowercase letters indicate significant differences among treatments (*P* < 0.05, one-way ANOVA followed by LSD test).

As shown in **Figure 2**, significant differences in seedling morphological traits were observed among different treatments. Compared with CK, exogenous GABA significantly promoted seedling growth, with SH (**Figure 2I**), SL (**Figure 2J**), RL (**Figure 2F**), LN (**Figure 2G**) and LRN (**Figure 2H**) respectively increased by 24.40%, 39.58%, 12.74%, 20.65%, and 78.35%. All stress treatments significantly inhibited seedling growth to varying degrees. Under S treatment, SH, SL, RL, LL, and LW decreased by 27.39%, 27.60%, 13.69%, 30.04%, and 33.48%, respectively (**Figures 2C, D**); under Cd treatment, the corresponding decreases were 19.70%, 16.79%, 27.96%, 27.85%, and 26.95%, respectively. The strongest inhibitory effects were observed under SCd treatment, with reductions of 39.75%, 37.99%, 37.13%, 42.65%, and 37.58%, respectively, indicating that combined stress exerted a stronger inhibitory effect on seedling morphological development. Compared with the corresponding stress treatments, exogenous GABA improved most morphological parameters. Compared with S treatment, all growth indices in SG increased by 13.26%–46.55%, with obvious improvements in LL, LW, SH, and SL(*P* < 0.05); all indices in CdG increased by 13.27%–57.14% compared with Cd, with notable recovery in SH and SL; growth indices in SCdG increased by 19.40%–66.67% compared with SCd, indicating that GABA effectively alleviated the inhibitory effects of combined stress on seedling morphological development. Notably, LRN under SCdG was slightly lower than that under SCd treatment, suggesting that the regulatory effects of GABA on different morphological traits were not entirely consistent.

### 3.2 Effects of exogenous GABA on physiological and biochemical parameters

**Figure 3:**
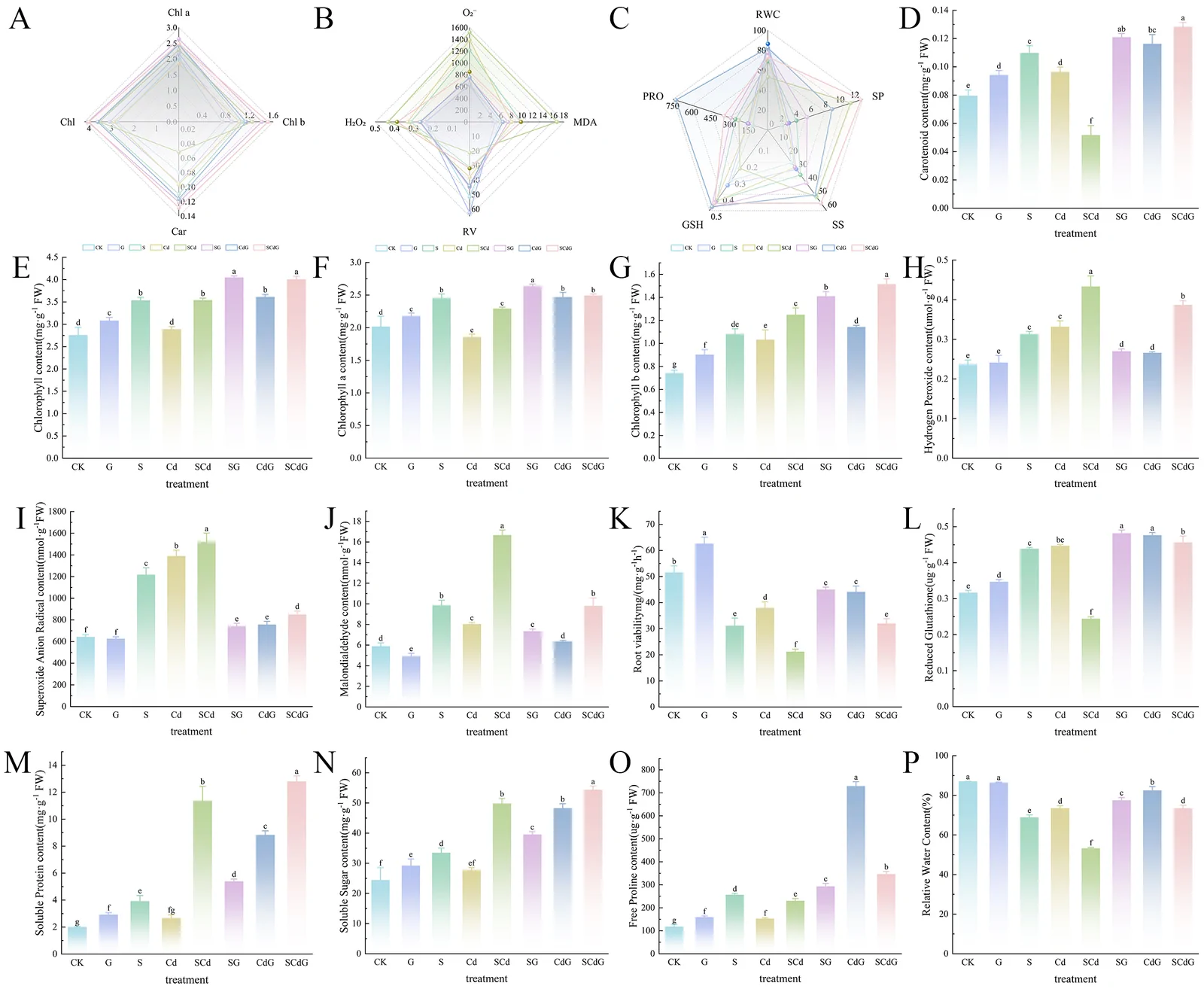
Effects of different treatments on photosynthetic pigments, oxidative damage, osmotic adjustment, root activity, and water status of *Prunus mongolica* seedlings. **(A)** is a radar plot of photosynthetic pigment-related parameters; **(B)** is a radar plot of oxidative damage indicators and RV; **(C)** is a radar plot of osmotic adjustment substances, RWC, and GSH. **(D)** is Car content; **(E)** is Chl content; **(F)** is Chl a content; **(G)** is Chl b content; **(H)** is H_2_O_2_ content; **(I)** is O_2_^-^ content; **(J)** is MDA content; **(K)** is RV; **(L)** is GSH content; **(M)** is SP content; **(N)** is SS content; **(O)** is Pro content; **(P)** is RWC. Different colors represent different treatments, and different lowercase letters indicate significant differences among treatments (*P* < 0.05, one-way ANOVA followed by LSD test). Except for RV, which was measured using root tissues, all other parameters shown in this figure were determined using leaf tissues.

**Figure 4:**
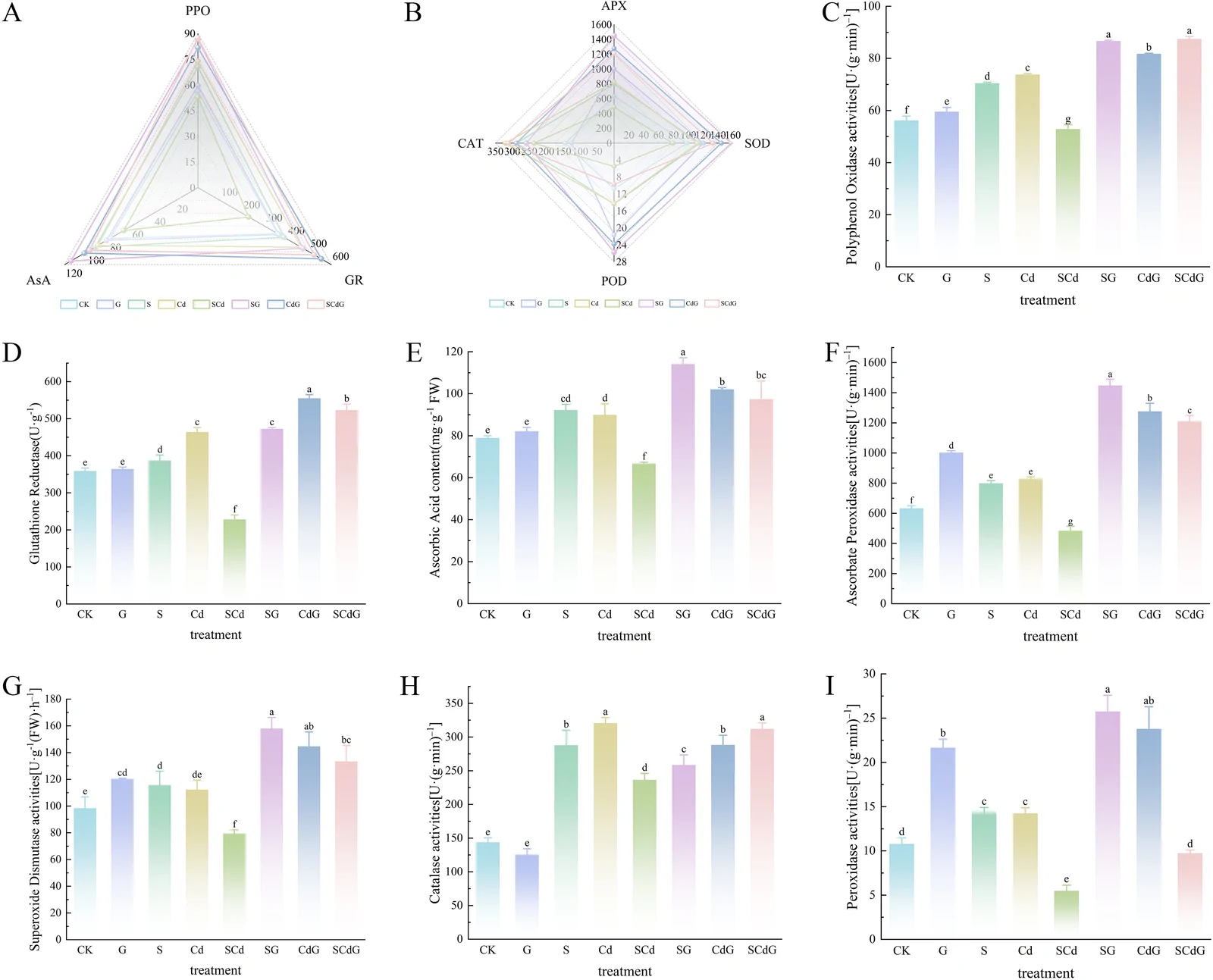
Effects of different treatments on antioxidant-related parameters of *Prunus mongolica* seedlings. **(A)** is a radar plot of PPO, GR, and AsA; **(B)** is a radar plot of APX, SOD, POD, and CAT; **(C)** is PPO activity; **(D)** is GR activity; **(E)** is AsA content; **(F)** is APX activity; **(G)** is SOD activity; **(H)** is CAT activity; **(I)** is POD activity. Different colors represent different treatments, and different lowercase letters indicate significant differences among treatments (*P* < 0.05, one-way ANOVA followed by LSD test). All antioxidant-related parameters shown in this figure were determined using leaf tissues.

The results in **Figures 3**, **4** showed that the redox balance, osmotic regulation status, and photosynthetic physiological processes of *Prunus mongolica* seedlings were significantly disrupted in S, Cd, and SCd, whereas exogenous GABA alleviated stress-induced damage by enhancing antioxidant defense capacity and osmotic adjustment ability. Compared with CK, GABA treatment significantly increased photosynthetic pigment content (**Figures 3D–G**), RV (**Figure 3K**), osmotic adjustment substances (**Figures 3M, O**), and the activities of several antioxidant enzymes (**Figure 4C, F, J, I**) (*P* < 0.05), indicating that GABA not only alleviated stress-induced damage but also potentially induced a certain degree of physiological pre-adaptation in seedlings even under non-stress conditions.

Under stress treatments, S, Cd, and SCd significantly increased the accumulation of O_2_^-^ (**Figure 3I**), H_2_O_2_ (**Figure 3H**), and MDA (**Figure 3J**) (*P* < 0.05). Among these, SCd exhibited the highest levels of all three indicators, with MDA showing the most pronounced increase (183.46%), indicating aggravated membrane lipid peroxidation under combined stress. Notably, SCd treatment significantly (*P* < 0.05) reduced Car content (**Figure 3D**), RV, and RWC (**Figure 3P**), whereas Chl a (**Figure 3F**), Chl b (**Figure 3G**), and Chl (**Figure 3E**) showed an overall increasing trend, suggesting a compensatory accumulation of chlorophyll-related traits under stress conditions. However, combined stress still exerted strong negative effects on Car maintenance, RV, and water retention capacity. Under S and Cd treatments, antioxidant enzyme activities, including SOD (**Figure 4G**), POD (**Figure 4J**), CAT (**Figure 4H**), APX (**Figure 4F**), PPO (**Figure 4C**), and GR (**Figure 4D**), were generally increased. In addition, non-enzymatic antioxidants such as AsA (**Figure 4E**) and GSH (**Figure 3L**) also increased at different levels, indicating that seedlings under single stress conditions activated both enzymatic and non-enzymatic antioxidant defense systems to scavenge ROS. Specifically, SOD primarily facilitated the dismutation of O_2_^-^, while CAT, APX, and POD were involved in scavenging of H_2_O_2_. The increases in AsA, GSH, and GR reflected the activation of the AsA–GSH cycle, contributing to cellular redox regulation. Meanwhile, increased accumulation of Pro, SS, and SP under stress conditions suggested improved osmotic adjustment, thereby contributing to the maintenance of cellular osmotic balance. However, under SCd stress treatment, although antioxidant and osmotic regulation systems were induced, ROS and MDA still accumulated at high levels, while RV and RWC remained significantly reduced (*P* < 0.05), suggesting that the endogenous defense capacity was insufficient to fully counteract the damage caused by combined stress.

Following exogenous GABA treatment, oxidative damage in SG, CdG, and SCdG groups was markedly alleviated, as evidenced by significant decreases in O_2_^-^, H_2_O_2_, and MDA contents (*P* < 0.05), while photosynthetic pigments, RV, and RWC were generally restored or maintained at relatively higher levels. Meanwhile, GABA further enhanced antioxidant enzyme activities (e.g., SOD) and the accumulation of osmotic adjustment substances, indicating improved ROS scavenging capacity and strengthened osmotic regulation, thereby maintaining cellular stability under stress. The results indicated that GABA exhibited stress type-dependent regulatory effects: under Cd treatment, the promotive effects of GABA on Pro accumulation and the activities of certain antioxidant enzymes were more pronounced; under SCd treatment, GABA more strongly enhanced SS, SP, and antioxidant system components, thereby alleviating oxidative damage and photosynthetic inhibition.

### 3.3 Multivariate statistical and correlation analysis of germination, morphological, and physiological traits

**Figure 5:**
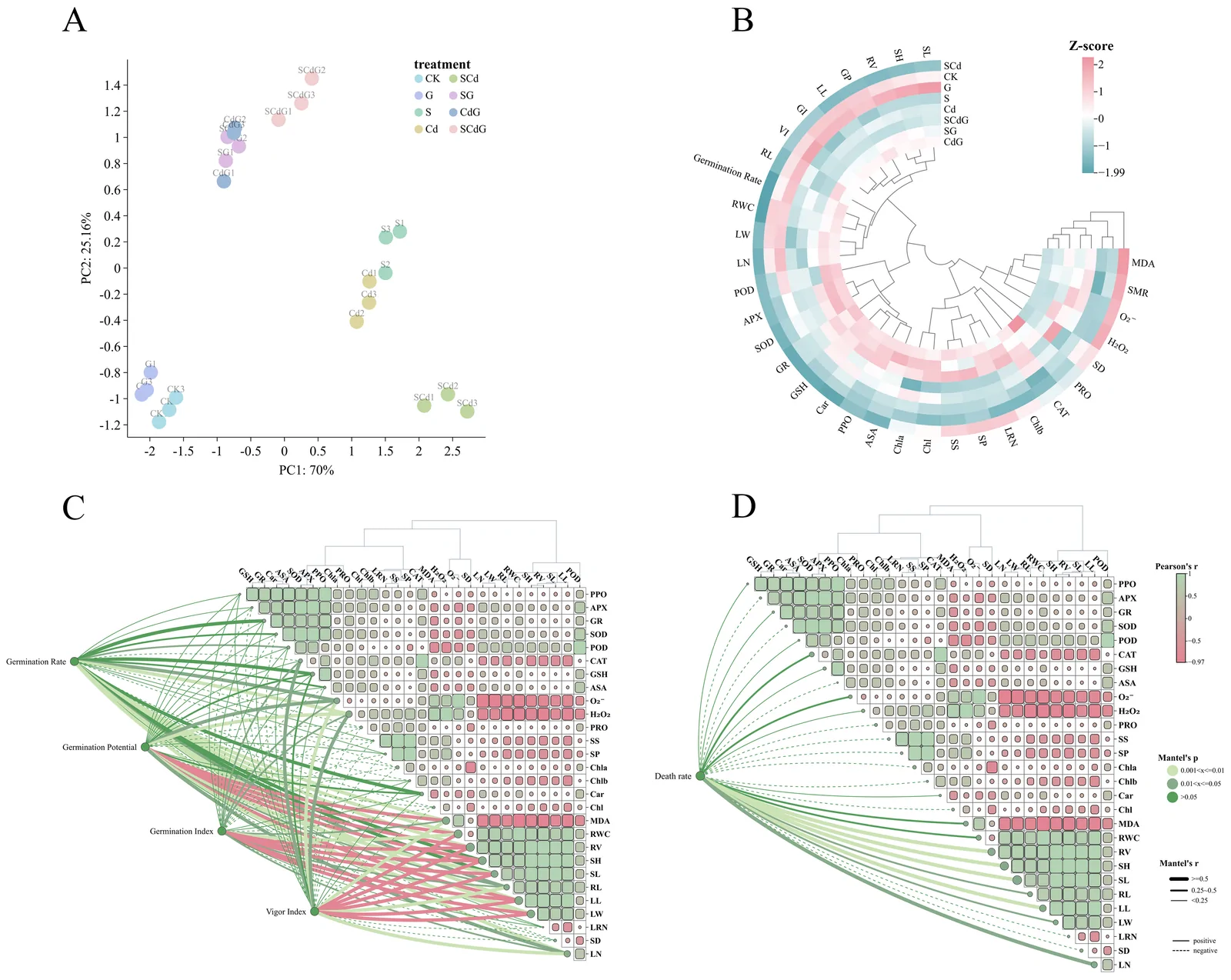
Multivariate statistical and correlation analyses of physiological, morphological, and germination traits. All data were normalized using Z-score transformation. **(A)** is PCA based on all measured traits; each point represents a biological replicate, and different colors indicate different treatments; **(B)** is a circular clustered heatmap of germination, morphological, and physiological and biochemical traits under different treatments. **(C)** is a Pearson correlation heatmap combined with Mantel test between germination-related traits and physiological/morphological traits. **(D)** is a Pearson correlation heatmap combined with Mantel test between death rate and physiological/morphological traits. In **(C, D)**, square color and size represent Pearson correlation coefficients; arcs indicate Mantel correlations between target traits and other measured variables; line width represents Mantel’s *r* value; line color indicates significance level; solid and dashed lines represent positive and negative correlations, respectively.

As shown in **Figure 5**, significant differences were observed among different treatments in seed germination, seedling growth, and physiological responses of *Prunus mongolica*. Principal component analysis (PCA, **Figure 5A**) showed that PC1 and PC2 explained 70.00% and 25.16% of the total variance, respectively, with clear separation among different treatments. The ring clustering heatmap (**Figure 5B**), based on standardized relative values, illustrated the overall distribution patterns of germination, morphological, and physiological indices of *Prunus mongolica* under different treatments. The results showed that oxidative damage-related indicators (O_2_^-^, H_2_O_2_, MDA, and DR) were generally elevated under stress conditions, with the most pronounced increases observed under SCd treatment. In contrast, antioxidant enzyme activities (SOD, CAT, POD, APX, GR, and GSH), osmotic adjustment substances (Pro, SS, and SP), and growth- and water-related indices (RWC, RV, GI, VI, and morphological traits) were generally reduced under stress conditions, with more severe decreases under SCd treatment. Following exogenous GABA treatments (SG, CdG, and SCdG), oxidative damage-related indicators were reduced, whereas antioxidant enzyme activities, osmotic regulation, and growth-related traits were partially restored to varying degrees.

Notably, SCdG treatment alleviated the accumulation of O_2_^-^ and MDA and partially mitigated growth inhibition under SCd stress, although H_2_O_2_ and certain water- and photosynthesis-related indices did not fully recover to control levels. Cluster analysis further revealed a clear separation between oxidative damage-related indicators and growth- and water-related traits, indicating distinct response modules under different treatments.

Pearson correlation analysis (**Figures 5C, D**) showed a significant positive correlation among oxidative damage-related indicators, including O_2_^-^, H_2_O_2_, and MDA. Among them, MDA exhibited a strong coupling relationship with O_2_^-^ (*r* = 0.79, *P* = 0.018) and H_2_O_2_ (*r* = 0.91, *P* = 0.002), indicating a highly consistent variation pattern between ROS accumulation and membrane lipid peroxidation. At the level of water status and growth traits, MDA showed significant negative correlations with RWC (*r* = −0.97, *P* < 0.001), RV (*r* = −0.90, *P* = 0.0027), and SH (*r* = −0.86, *P* = 0.0063), suggesting that enhanced oxidative damage was closely associated with reduced water status and inhibited growth. Within the antioxidant system, clear synergistic relationships were observed. SOD was positively correlated with POD (*r* = 0.81, *P* = 0.014), APX with GR (*r* = 0.82, *P* = 0.013), and the strongest correlation was observed between SOD and APX (*r* = 0.98, *P* < 0.001), indicating a highly coordinated response among antioxidant enzyme system. In addition, non-enzymatic antioxidants such as AsA and GSH showed overall positive correlations with enzymatic antioxidant components, together forming an integrated ROS scavenging network.

In contrast, Mantel test analysis (**Figures 5C, D**) further revealed cross-level association patterns among germination, morphological, and physiological traits of *Prunus mongolica*. Within germination-related traits, germination rate showed significant correlations with water status indicators, including RWC (*r* = 0.89, *P* = 0.003) and RV (*r* = 0.69, *P* = 0.004), as well as root-related trait RL (*r* = 0.51, *P* = 0.015). It also exhibited strong associations with oxidative damage indicators, including MDA (*r* = 0.98, *P* = 0.002) and H_2_O_2_ (*r* = 0.71, *P* = 0.005), indicating that seed germination progression was collectively associated with water maintenance capacity and oxidative stress levels. At the seedling vigor level, VI showed significant positive correlations with both aboveground and belowground growth traits, including SH (*r* = 0.92, *P* = 0.001), RV (*r* = 0.94, *P* = 0.001), SL (*r* = 0.83, *P* = 0.001), and RL (*r* = 0.65, *P* = 0.008), suggesting coordinated regulation between root function and shoot growth in determining overall seedling vigor. In contrast, death rate exhibited significant associations with both morphological and root-related traits, including SH (*r* = 0.90, *P* = 0.002), SL (*r* = 0.98, *P* = 0.002), RV (*r* = 0.78, *P* = 0.005), and RL (*r* = 0.45, *P* = 0.026), indicating that variation in mortality was closely associated with growth inhibition and reduced root functional capacity.

### 3.4 Transcriptome sequencing and data quality assessment

**Figure 6:**
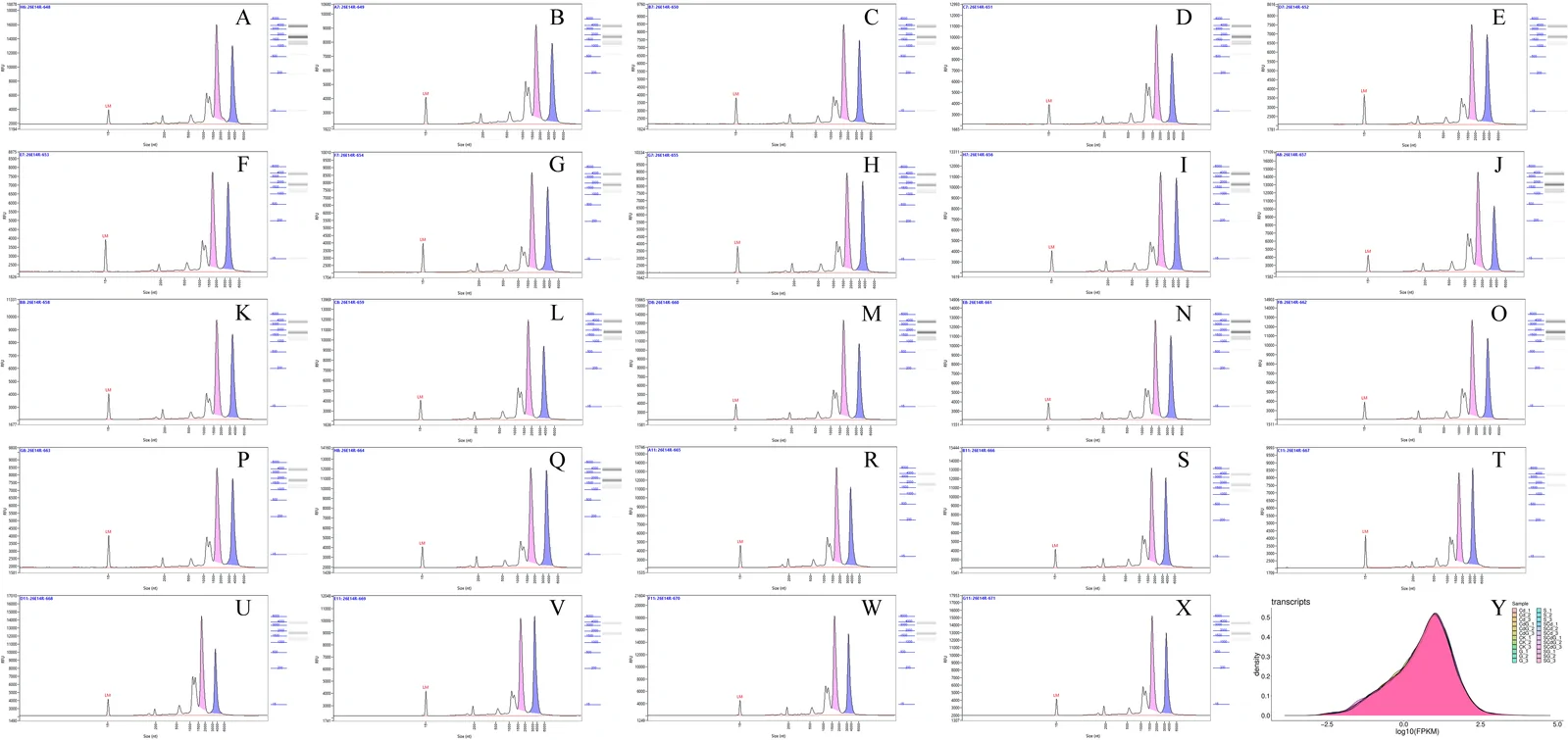
RNA quality control profiles and density distribution of gene expression levels. **(A–X)** are the 5400 Fragment Analyzer automated electrophoresis quality control profiles of RNA from different treatment groups. The x-axis represents RNA fragment size (nt), and the y-axis represents fluorescence signal intensity relative fluorescence units (RFU). LM is the lower marker peak. The black curve represents the distribution of sample RNA fragments. The pink and blue regions represent the major rRNA peak regions, respectively. The panel on the right shows the corresponding virtual gel image. **(A)** is CK_1; **(B)** is CK_2; **(C)** is CK_3; **(D)** is G_1; **(E)** is G_2; **(F)** is G_3; **(G)** is S_1; **(H)** is S_2; **(I)** is S_3; **(J)** is Cd_1; **(K)** is Cd_2; **(L)** is Cd_3; **(M)** is SCd_1; **(N)** is SCd_2; **(O)** is SCd_3; **(P)** is SG_1; **(Q)** is SG_2; **(R)** is SG_3; **(S)** is CdG_1; **(T)** is CdG_2; **(U)** is CdG_3; **(V)** is SCdG_1; **(W)** is SCdG_2; and **(X)** is SCdG_3. **(Y)** is the density distribution plot of transcript expression levels in samples under different treatments. The x-axis represents log_10_ (FPKM), and the y-axis represents transcript expression density. Different colors represent different samples.

As shown in **Supplementary Tables 4** and **5**, total RNA quality and purity were evaluated using a NanoDrop ND-1000 spectrophotometer, and RNA integrity was further assessed using an Agilent Bioanalyzer 2100. All samples met the quality requirements for downstream analysis, with RNA concentration >50 ng/μL, RIN values >7.0, and total RNA amount >1 μg. RNA integrity profiles of 24 samples (8 treatments with 3 biological replicates each) were further validated using LabChip systems and the 5300/5400 Fragment Analyzer (**Figures 6A–X**). As shown in the electropherogram profiles, all samples exhibited high RNA integrity without obvious degradation.

To investigate the molecular responses of *Prunus mongolica* to S, Cd, and SCd under GABA treatment, transcriptome sequencing was performed on leaf samples from different treatments. Paired-end sequencing (PE150) was conducted using the Illumina NovaSeq™ X Plus platform following standard protocols. A total of 976.38 million raw reads were generated. After stringent quality control, including removal of low-quality reads, adapter sequences, and ambiguous bases, 140.82 Gb of clean data were obtained. To evaluate the randomness and quality of library construction, read distribution across transcript regions was analyzed. As shown in **Figure 6Y**, the distribution of mapped reads across transcript regions followed a typical Gaussian pattern, and the regression curve showed a relatively smooth slope, indicating high-quality library construction suitable for downstream differential expression analysis. A total of 23,798 transcripts were obtained. Among them, 23,798 transcripts (100.00%) were successfully annotated in the NR database. Annotation rates were 99.00% in NOG (23,561 transcripts), 90.49% in Pfam (21,535), 76.39% in Swiss-Prot (18,180), 74.53% in GO (17,736), 48.01% in COG (11,426), and 22.41% in KEGG (5,334), respectively.

### 3.5 DEGs screening, identification, and comparative analysis

**Figure 7:**
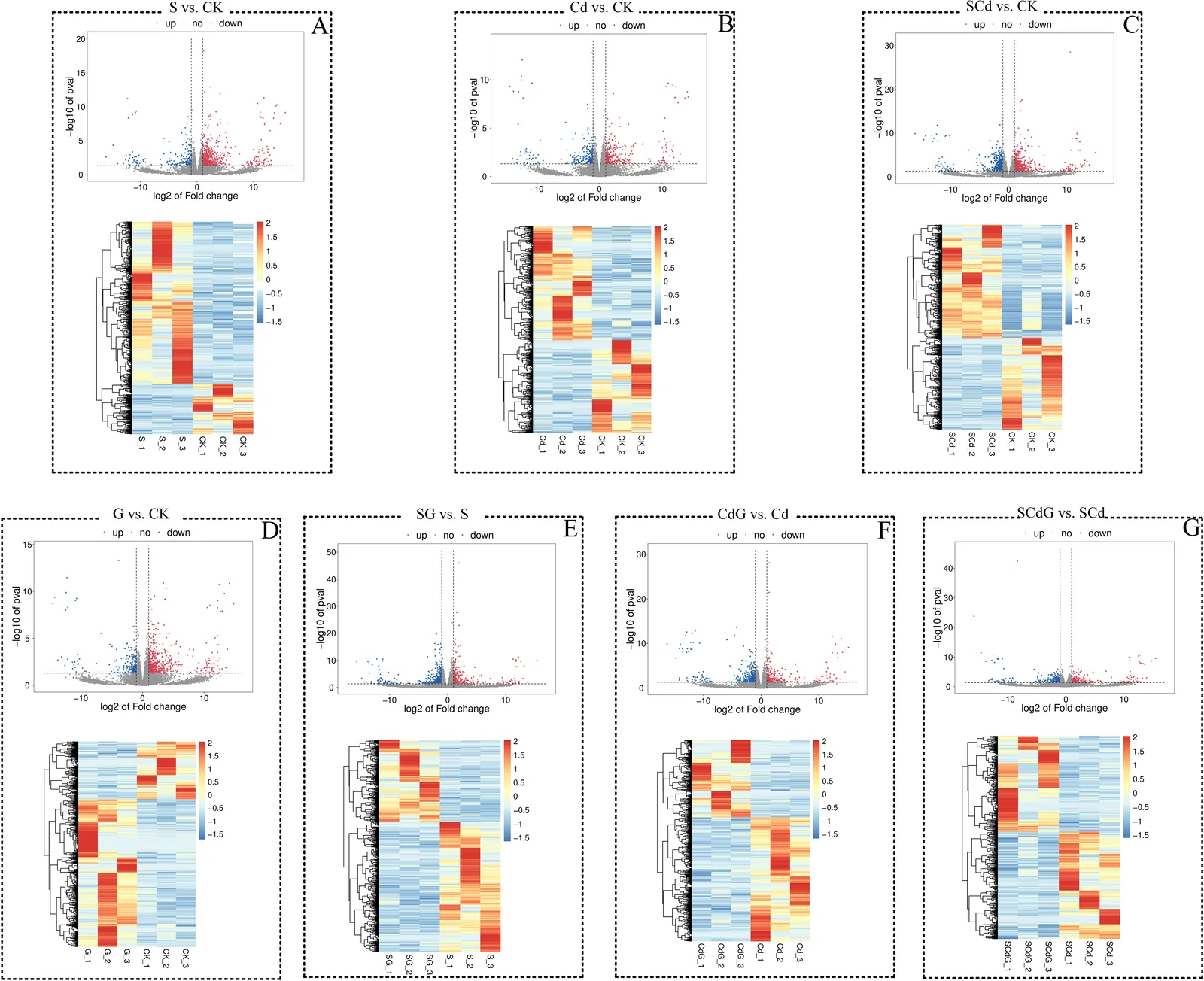
Volcano plots and hierarchical clustering heatmaps of DEGs in different comparison groups. **(A)** is S vs. CK; **(B)** is Cd vs. CK; **(C)** is SCd vs. CK; **(D)** is G vs. CK; **(E)** is SG vs. S; **(F)** is CdG vs. Cd; and **(G)** is SCdG vs. SCd. In the volcano plots, red dots indicate significantly upregulated genes, blue dots indicate significantly downregulated genes, and gray dots indicate genes with no significant differential expression.

To evaluate the distribution characteristics, expression patterns, and consistency among biological replicates of differentially expressed genes (DEGs) across different treatments, volcano plot and hierarchical clustering analyses were performed for the seven comparison groups (**Figure 7**). The results showed that different numbers of DEGs were identified among the comparison groups. Specifically, 788, 570, 996, 735, 752, 617, and 495 DEGs were identified in S vs. CK, Cd vs. CK, SCd vs. CK, G vs. CK, SG vs. S, CdG vs. Cd, and SCdG vs. SCd, respectively. Among these comparisons, SCd vs. CK contained the largest number of DEGs, including 549 up-regulated and 447 down-regulated genes, whereas SCdG vs. SCd contained the fewest DEGs, including 233 up-regulated and 262 down-regulated genes. These results indicated that SCd induced the strongest transcriptomic response, while the transcriptional changes induced by GABA under combined stress were relatively lower than those observed under single-stress backgrounds.

**Figure 8:**
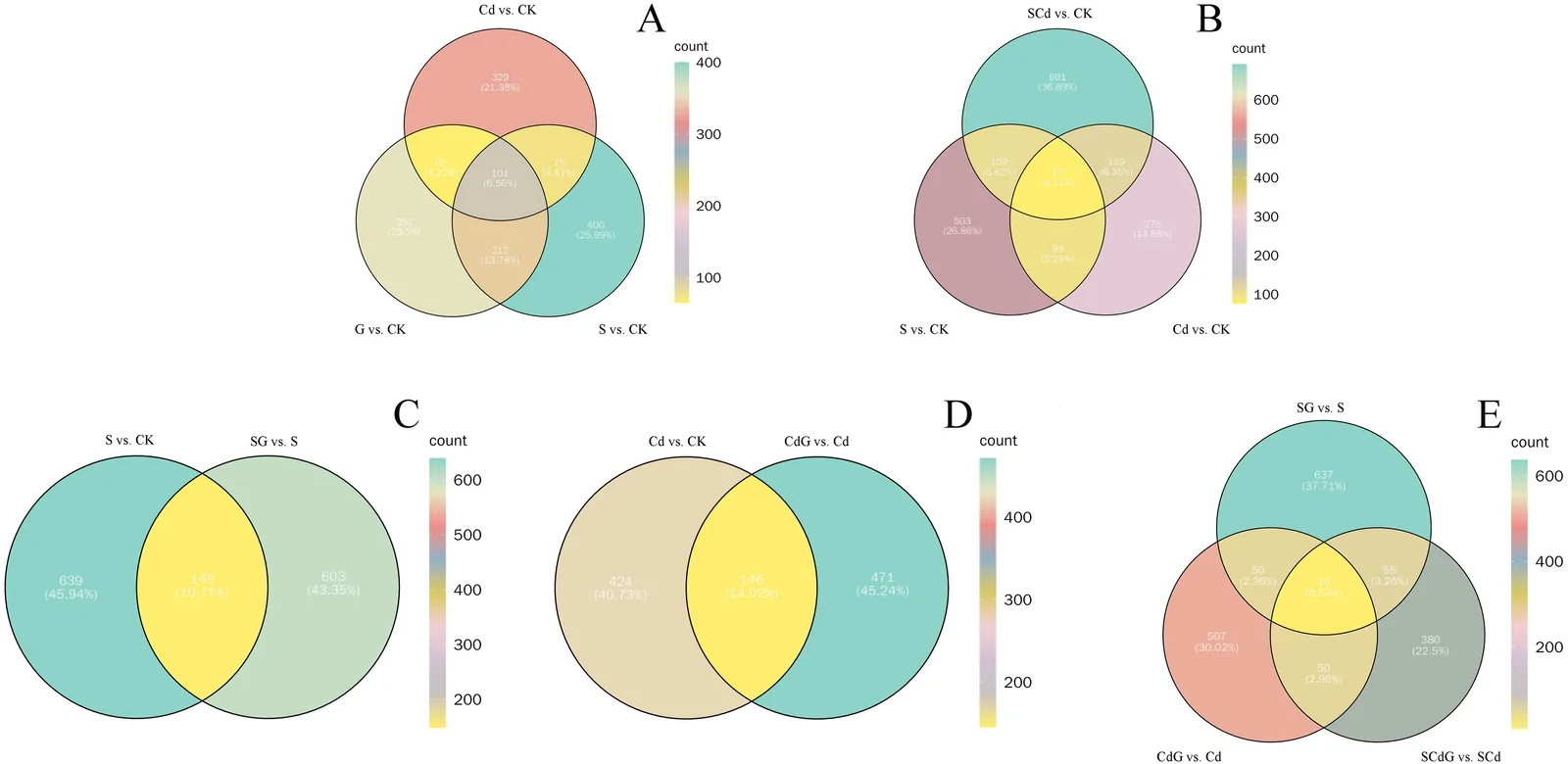
Venn diagrams showing overlap relationships of DEGs among different comparison groups. **(A)** is the Venn diagram of Cd vs. CK, S vs. CK, and G vs. CK; **(B)** is the Venn diagram of SCd vs. CK, Cd vs. CK, and S vs. CK; **(C)** is the Venn diagram of SG vs. S and S vs. CK; **(D)** is the Venn diagram of CdG vs. Cd and Cd vs. CK; **(E)** is the Venn diagram of SG vs. S, CdG vs. Cd, and SCdG vs. SCd. Each circle represents a differential comparison group, and the numbers within the circles indicate the number of DEGs in unique or overlapping regions among the comparison groups.

To further compare the overlap of DEGs among different treatments, the seven comparison groups were classified according to the analytical objectives, and Venn diagrams were constructed (**Figure 8**). **Figure 8A** compared DEGs between single-factor treatments and CK, including Cd vs. CK, S vs. CK, and G vs. CK, showing 329, 400, and 357 unique DEGs, respectively, with 101 DEGs shared among the three comparison groups. **Figure 8B** compared DEGs between combined stress and single stress responses, including SCd vs. CK, Cd vs. CK, and S vs. CK, revealing 691 unique DEGs in SCd vs. CK, while only 77 DEGs were shared among the three comparison groups. **Figures 8C, D** respectively focused on GABA-responsive DEGs under S and Cd backgrounds, revealing 603 unique DEGs in SG vs. S and 471 unique DEGs in CdG vs. Cd. **Figure 8E** further compared the common characteristics of GABA-responsive DEGs under different stress backgrounds, including SG vs. S, CdG vs. Cd, and SCdG vs. SCd, revealing only 10 shared DEGs among the three comparison groups, whereas the number of DEGs shared between any two GABA-related stress comparisons was higher than that shared among all three groups. These results indicated that GABA-mediated transcriptional responses were largely stress-specific rather than uniform across different stress backgrounds.

### 3.6 Functional annotation and enrichment analysis of DEGs

**Figure 9:**
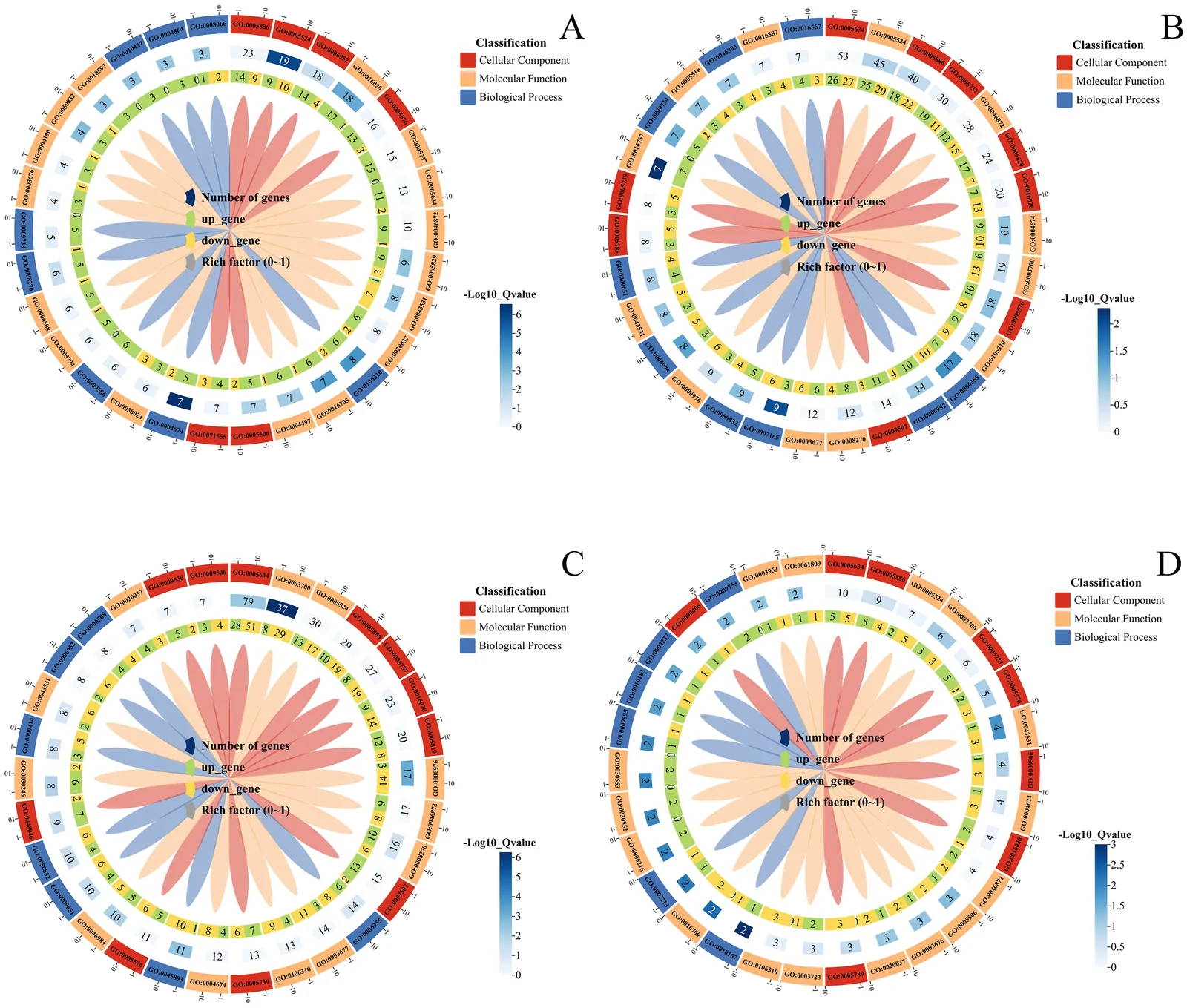
GO functional enrichment analysis of DEGs. **(A)** is the GO functional enrichment analysis of DEGs in the S vs. CK group; **(B)** is the GO functional enrichment analysis of DEGs in the Cd vs. CK group; **(C)** is the GO functional enrichment analysis of DEGs in the SG vs. S group; **(D)** is the GO functional enrichment analysis of DEGs in the SCd vs. CK group. Red represents cellular component; orange represents molecular function; blue represents biological process; green represents the number of upregulated DEGs in each GO term; yellow represents the number of downregulated DEGs in each GO term; GO IDs correspond to the unique identifiers of each functional term.

**Figure 10:**
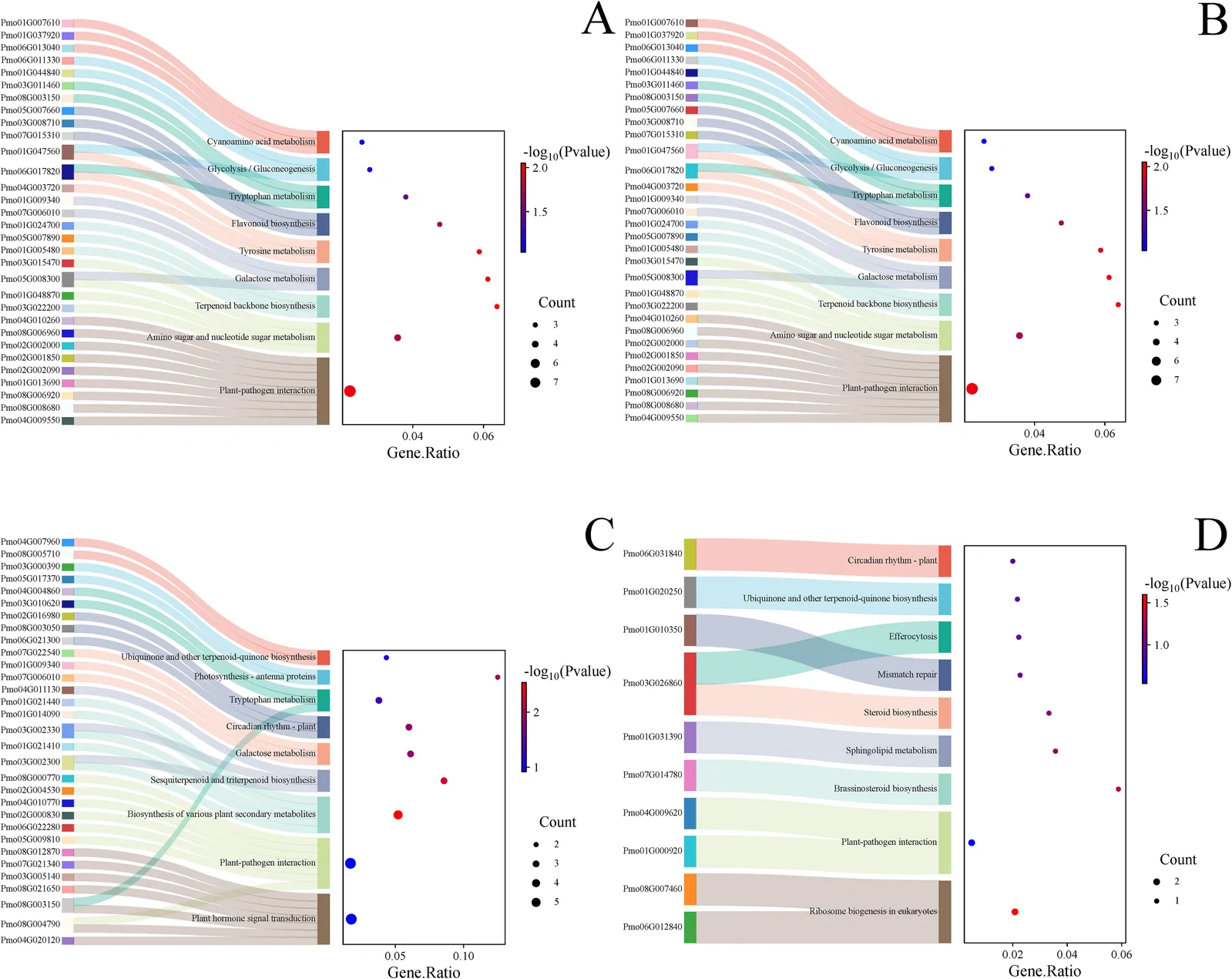
KEGG functional enrichment analysis of DEGs. **(A)** is the KEGG functional enrichment analysis of DEGs in the S vs. CK group; **(B)** is the KEGG functional enrichment analysis of DEGs in the Cd vs. CK group; **(C)** is the KEGG functional enrichment analysis of DEGs in the SG vs. S group; **(D)** is the KEGG functional enrichment analysis of DEGs in the SCd vs. CK group. Different colors on the left represent different KEGG pathways and corresponding DEGs, while the right panel shows the KEGG pathway enrichment results of DEGs.

GO functional enrichment analysis of DEGs across the seven comparison groups showed that multiple functional terms were annotated in all groups (**Supplementary Table 6**). In Cd vs. CK, a total of 169 GO terms were identified, belonging to Biological Process (79), Cellular Component (27), and Molecular Function (63). Notably, SG vs. S showed the highest number of annotated GO terms, with 1,341 DEGs mapped to 586 GO terms, indicating the most extensive functional annotation among all comparison groups. KEGG enrichment analysis of DEGs revealed that multiple biological pathways were significantly annotated across all comparison groups (**Supplementary Table 7**). SCd vs. CK exhibited the highest number of enriched pathways (49 pathways), involving Cellular Processes, Environmental Information Processing, Genetic Information Processing, Metabolism, and Organismal Systems. In GABA-treated groups, SG vs. S showed 32 enriched pathways, CdG vs. Cd showed 24 pathways, and SCdG vs. SCd showed only 16 pathways, indicating a gradual reduction in pathway-level responses under more complex stress conditions. Considering the differences in DEG numbers among comparison groups, four representative groups (S vs. CK, SCd vs. CK, SG vs. S, and SCdG vs. SCd) were selected for GO and KEGG enrichment analyses (**Figures 9**, **10**), corresponding to single-stress response mechanisms, combined-stress specificity, GABA-mediated regulation under single salt stress, and GABA-mediated response under combined stress, respectively. Other comparison groups were provided in **Supplementary Figure 1** to maintain analytical completeness and clarity.

To further investigate the mechanisms underlying single stress, GO enrichment analysis of S vs. CK showed that 655 DEGs were annotated to 344 GO terms (**Figure 9A**). The top 30 enriched GO terms included “plasma membrane”, “ATP binding”, and “defense response”, with 14 up- and 9 down-regulated DEGs, 9 up- and 10 down-regulated DEGs, and 14 up- and 4 down-regulated DEGs, respectively. In SCd vs. CK (**Figure 9B**), a total of 996 DEGs were analyzed, and the most significantly enriched terms included “plasma membrane” (18 up- and 22 down-regulated DEGs) and “ATP binding” (25 up- and 20 down-regulated DEGs). SG vs. S, used as a representative of the mechanism by which exogenous GABA alleviated single salt stress in this study, yielded 1,341 DEGs annotated to 586 GO terms, among which “ATP binding” (13 upregulated and 17 downregulated DEGs) ranked among the top three enriched functional categories (**Figure 9C**). In SCdG vs. SCd (**Figure 9D**), only 6 DEGs were annotated, mainly enriched in “ATP binding” (3 up- and 3 down-regulated DEGs).

In this study, DEGs were ranked in descending order according to the number of genes annotated to KEGG pathways in each comparison group, and the top nine enriched pathways are shown in **Figure 10**. In the S vs. CK comparison group, KEGG functional annotation revealed 62 upregulated and 14 downregulated DEGs, and the top nine pathways shown in **Figure 10A** collectively involved 52 DEGs, with “Plant–pathogen interaction” containing the largest number of DEGs. In the SCd vs. CK comparison group (**Figure 10B**), 110 DEGs were annotated to 49 KEGG pathways, with “Plant–pathogen interaction” remaining the most enriched pathway, containing 5 upregulated and 4 downregulated DEGs. In the SG vs. S comparison group (**Figure 10C**), both “Plant hormone signal transduction” and “Plant–pathogen interaction” pathways contained 7 DEGs each, showing slight differences compared with the above two comparison groups. The SCdG vs. SCd comparison group (**Figure 10D**), yielded 17 DEGs annotated to 16 KEGG pathways, which was only higher than Cd vs. CK (11 DEGs) among all comparison groups (**Supplementary Figure 2**).

### 3.7 qRT-PCR validation of DEGs

**Figure 11:**
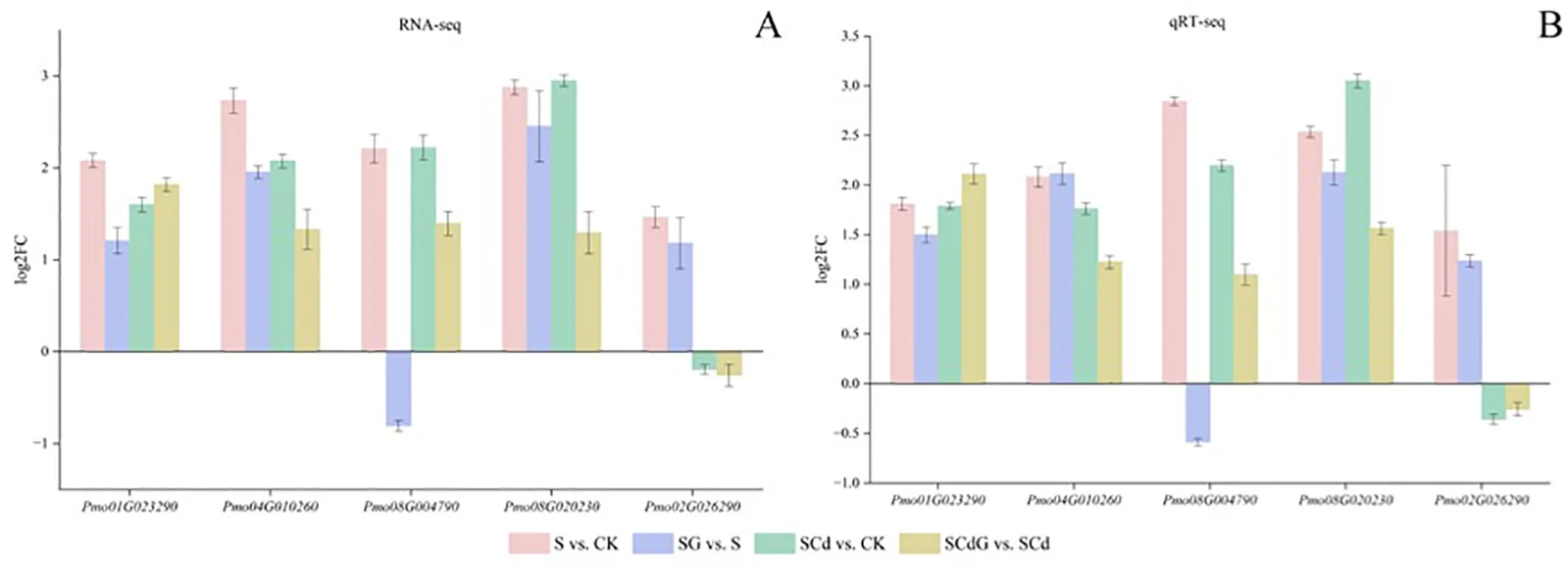
qRT-PCR validation of gene expression results. **(A)** is RNA-seq validation; **(B)** is qRT-seq validation. The x-axis represents five candidate genes, and the y-axis represents log_2_FC. Different colors indicate different comparison groups (S vs. CK, SG vs. S, SCd vs. CK, and SCdG vs. SCd), and error bars represent standard errors of biological replicates.

To validate the RNA-seq results and further confirm the expression patterns of DEGs, five representative DEGs were randomly selected for qRT-PCR analysis (**Figure 11**). These genes exhibited different expression patterns across the S vs. CK, SG vs. S, SCd vs. CK, and SCdG vs. SCd comparison groups. Among them, *Pmo01G023290* (K00480) showed a consistently up-regulated expression pattern across all four comparison groups. In contrast, *Pmo04G010260* (K04626) was significantly up-regulated in S vs. CK, SCd vs. CK, and SCdG vs. SCd, but exhibited a down-regulated expression trend in SG vs. S. Overall, the expression trends of the five selected DEGs obtained from qRT-PCR were consistent with those from RNA-seq analysis (**Figure 11**), confirming the reliability of the transcriptome sequencing data. Under alkali salt, cadmium, and combined stress conditions, these genes showed significant expression changes, while exogenous GABA treatment markedly modulated their expression levels. These results suggest that GABA may be involved in stress mitigation by regulating pathways associated with Ca^2+^ signaling (K04626/K04016), MAPK cascade signaling (K04016), and ROS metabolism (K04016).

### 3.8 GABA-induced transcription factor analysis

Transcription factor (TFs) is key regulators that control downstream gene expression during plant responses to abiotic stresses, playing essential roles in signal transduction and “defense response” under S, Cd, and SCd conditions. The aforementioned DEGs analysis and qRT-PCR validation confirmed the high reliability of the transcriptome data. Therefore, differentially expressed transcription factors were further identified to elucidate the transcriptional regulatory mechanisms mediated by exogenous GABA in *Prunus mongolica* under stress conditions.

**Figure 12:**
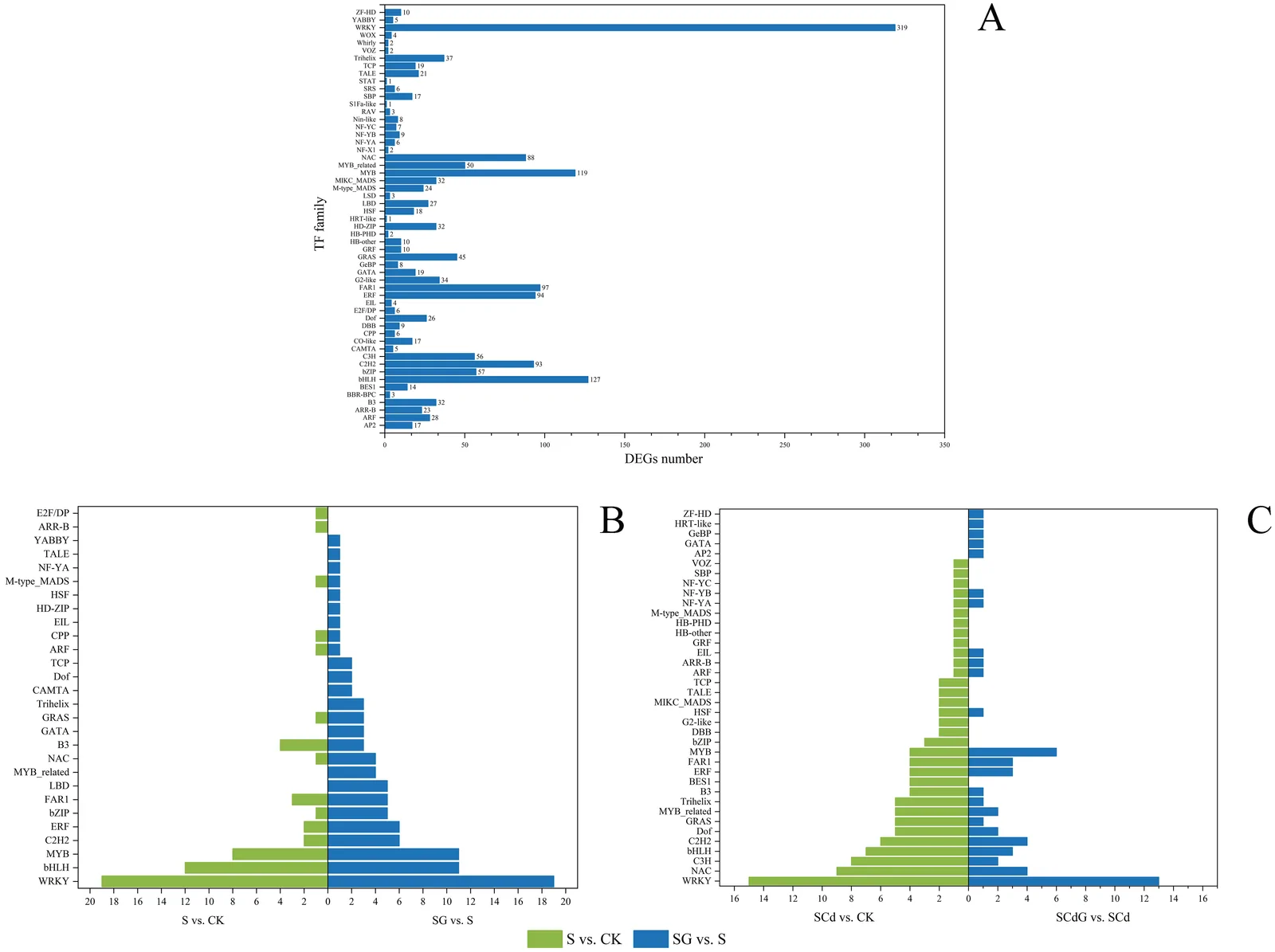
TF family analysis of DEGs. **(A)** is the classification and abundance distribution of TF families identified from DEGs. **(B)** is the comparison of TF family distribution between S vs. CK and SG vs. S. **(C)** is the comparison of TF family distribution between SCd vs. CK and SCdG vs. SCd. Blue bars represent the number of upregulated DEGs, and green bars represent the number of downregulated DEGs in each TF family.

As shown in **Figure 12A**, a total of 1,716 differentially expressed transcription factors (DE-TFs) were identified, belonging to 55 TF families. Among them, the most abundant families were WRKY (18.6%), bHLH (7.4%), and MYB (6.9%), indicating that these families play central regulatory roles in stress responses. To further explore the regulatory effects of GABA under different stress conditions, comparisons between S vs. CK and SG vs. S were conducted (**Figure 12B**). The results showed that 13 TF families were specifically identified in SG vs. S but not in S vs. CK, including LBD, MYB_related, GATA, Trihelix, CAMTA, Dof, TCP, EIL, HD-ZIP, HSF, NF-YA, TALE, and YABBY, suggesting that GABA treatment induces additional transcriptional regulatory networks under salt stress. Under cadmium-related conditions (**Figure 12C**), comparison between SCd vs. CK and SCdG vs. SCd revealed distinct TF profiles. A total of 56 DEGs were annotated as transcription factors in SCdG vs. SCd, distributed across 24 TF families. Notably, AP2, GATA, GeBP, HRT-like, and ZF-HD were uniquely identified in this treatment, indicating that GABA may regulate stress adaptation under combined stress by activating specific TF families.

### 3.9 GABA-induced KEGG pathway analysis

**Figure 13:**
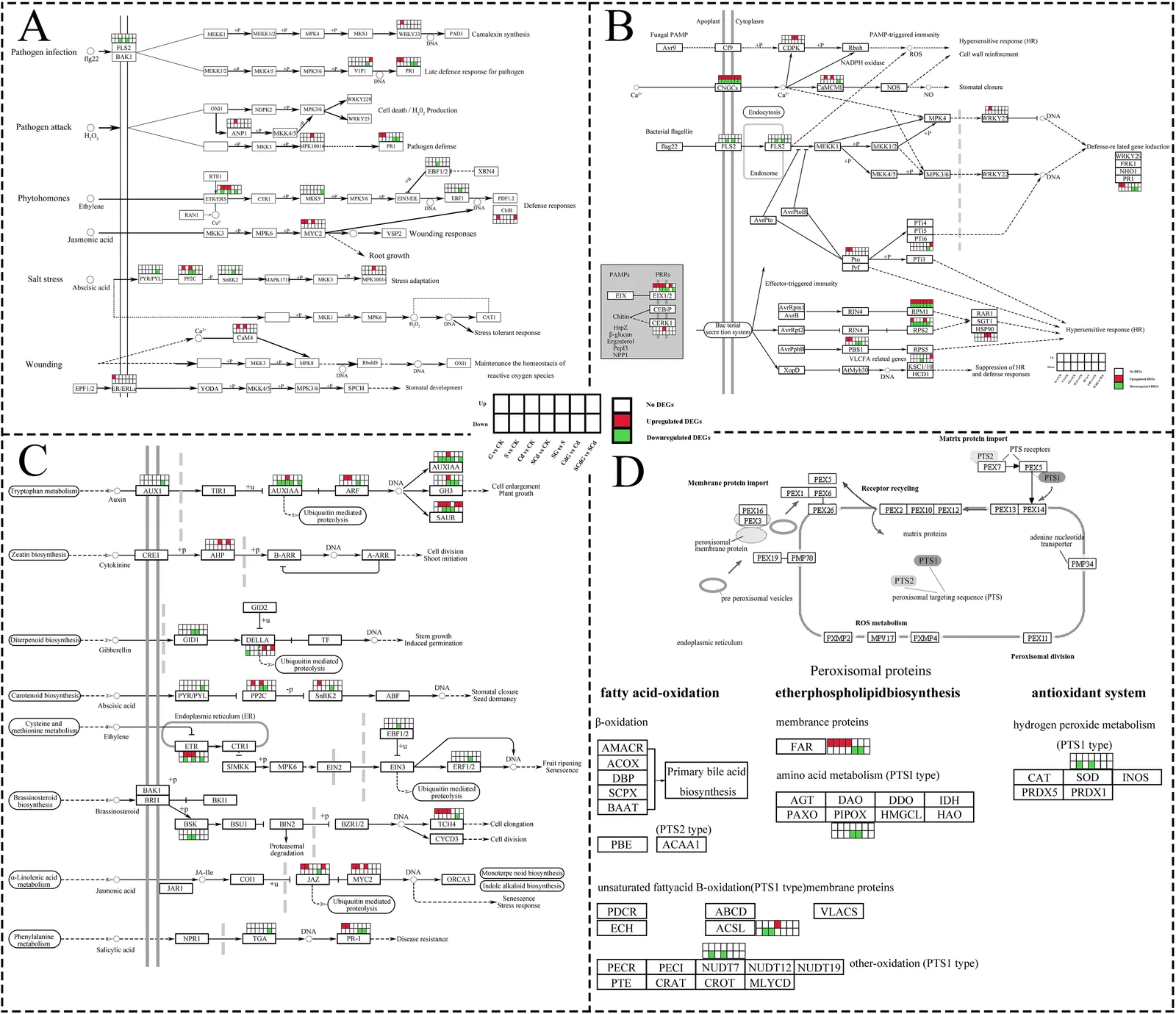
KEGG pathway maps of DEGs in different comparison groups. **(A)** shows the “MAPK signaling pathway–plant” with differentially expressed genes mapped onto the pathway; **(B)** shows the “Plant–pathogen interaction” pathway with differentially expressed genes mapped under different treatments; **(C)** shows the “Plant hormone signal transduction” pathway with differentially expressed genes mapped under different treatments; **(D)** shows the “Peroxisome” pathway with differentially expressed genes mapped onto the pathway under different treatments. Red indicates upregulated genes, and green indicates downregulated genes under different treatments.

The above GO and KEGG enrichment results indicated that exogenous GABA treatment significantly affected the functional enrichment patterns of DEGs in *Prunus mongolica* under S, Cd, and SCd conditions. The number of KEGG pathways enriched in G vs. CK, S vs. CK, Cd vs. CK, and SCd vs. CK was 40, 44, 11, and 49, respectively, with SCd vs. CK showing the highest number of enriched pathways, suggesting that combined stress induced a broader transcriptional response than single stresses. In the GABA-treated groups, SG vs. S, CdG vs. Cd, and SCdG vs. SCd were enriched in 32, 24, and 16 KEGG pathways, respectively, indicating that the regulatory effects of GABA varied among different stress backgrounds. To highlight pathways most closely associated with the observed phenotypic and physiological responses, further analysis focused on “MAPK signaling pathway–plant” (**Figure 13A**), “Plant–pathogen interaction” (**Figure 13B**), “Plant hormone signal transduction” (**Figure 13C**), and “Peroxisome” (**Figure 13D**).

In the “MAPK signaling pathway–plant” (**Figure 13A**), S vs. CK showed up-regulation of DEGs related to *PR1*/*STS14*, *MYC2*/bHLH transcription factors, *WRKY33*, histidine kinase-like protein, and *PP2C*, indicating activation of stress signal perception, JA/ABA-related signaling, and defense-associated transcriptional regulation. These changes were consistent with the increased O_2_^-^, H_2_O_2_, and MDA accumulation, reduced RWC, decreased photosynthetic pigment contents, and inhibited seedling growth under S treatment. In SG vs. S, several MAPK-related DEGs, including *PP2C*, *PR1*, *ERF1B*, LRR receptor-like protein kinase, and serine/threonine-protein kinase genes, were down-regulated, suggesting that GABA may attenuate excessive defense and hormone signaling under S stress. Under SCd stress, DEGs related to *NTF3*, *MYC3*, *PP2C*, EIN3-binding F-box protein, chitinase-like protein, and calmodulin-like protein 11 indicated a more complex signaling response involving MAPK cascade, Ca^2+^ signaling, and JA/ABA/ethylene-related pathways. In SCdG vs. SCd, the differential regulation of basic chitinase, bZIP61-like, and MKK9 suggested that GABA may contribute to combined-stress mitigation by modulating MAPK-mediated defense signaling.

In the “Plant–pathogen interaction” pathway (**Figure 13B**), DEGs were mainly associated with CNGCs, CaM-like proteins, CDPKs, serine/threonine-protein kinases, LRR receptor-like protein kinases, WRKY33, PR1/STS14, and RGA/RPP-type resistance proteins. These genes are closely related to Ca^2+^ signaling, defense responses, and ROS-associated signal transduction. Under S and SCd treatments, the differential expression of these genes coincided with enhanced ROS accumulation, membrane lipid peroxidation, and growth inhibition, suggesting that this pathway participated in stress-induced defense activation and oxidative damage responses. After GABA treatment, many CNGC, LRR-RLK, and RGA/RPP-related genes were down-regulated in SG vs. S and SCdG vs. SCd, whereas ethylene-responsive transcription factor *CRF2* and 3-ketoacyl-CoA synthase 19 were up-regulated. These results suggest that GABA may help rebalance overactivated defense signaling and ROS-related responses, thereby contributing to improved membrane stability and stress recovery.

In the “Plant hormone signal transduction” pathway (**Figure 13C**), DEGs were mainly involved in auxin, ABA, JA, ethylene, and GA-related signaling. Under S stress, genes related to IAA/ARF, *PP2C*, *MYC2*, TIFY, CaM-like proteins, *PR1*, and cell wall loosening-related proteins were differentially expressed, indicating that S treatment affected both growth-related and defense-related hormone signaling. This was consistent with the observed growth inhibition, ROS accumulation, and water imbalance. Following GABA treatment, SG vs. S showed extensive regulation of auxin-, ABA-, ethylene-, JA-, and defense-related genes, suggesting that GABA may alleviate stress damage by reprogramming hormone-mediated growth–defense balance. Under SCd stress, DEGs involving IAA/SAUR/GH3, PP2C, MYC3, CDPK, EIN3-binding F-box protein, CaM-like protein 11, and cell wall-related proteins indicated stronger hormonal crosstalk. In SCdG vs. SCd, DEGs associated with DELLA protein RGL1-like, SAURs, and IAA2-like genes suggested that GABA may promote growth recovery under combined stress by modulating GA and auxin related signaling.

In the “Peroxisome” pathway (**Figure 13D**), DEGs were mainly related to fatty acid metabolism, redox homeostasis, and ROS scavenging. Under S stress, the differential expression of genes related to fatty acid reduction, long-chain acyl-CoA synthesis, nudix hydrolase, and SOD suggested that S treatment affected peroxisome-mediated lipid metabolism and ROS detoxification, which was consistent with increased MDA accumulation and membrane damage. Under SCd stress, the up-regulation of long-chain acyl-CoA synthetase 8 and fatty acid reductase 5, together with the down-regulation of nudix hydrolase 11-like protein and SOD-related gene, indicated that combined stress simultaneously affected lipid metabolism, membrane stability, and peroxisomal redox regulation. After GABA treatment, changes in peroxisome-related genes suggested that GABA may participate in maintaining redox balance and membrane stability by regulating peroxisome-associated lipid metabolism and ROS scavenging. Overall, these results indicate that GABA-mediated stress mitigation in *Prunus mongolica* is closely associated with coordinated regulation of “MAPK signaling pathway–plant”, defense-related Ca^2+^ signaling, hormone signal transduction, and peroxisome-mediated ROS metabolism.

### 3.10 Response characteristics of KEGG-GSEA under exogenous GABA treatment

**Figure 14:**
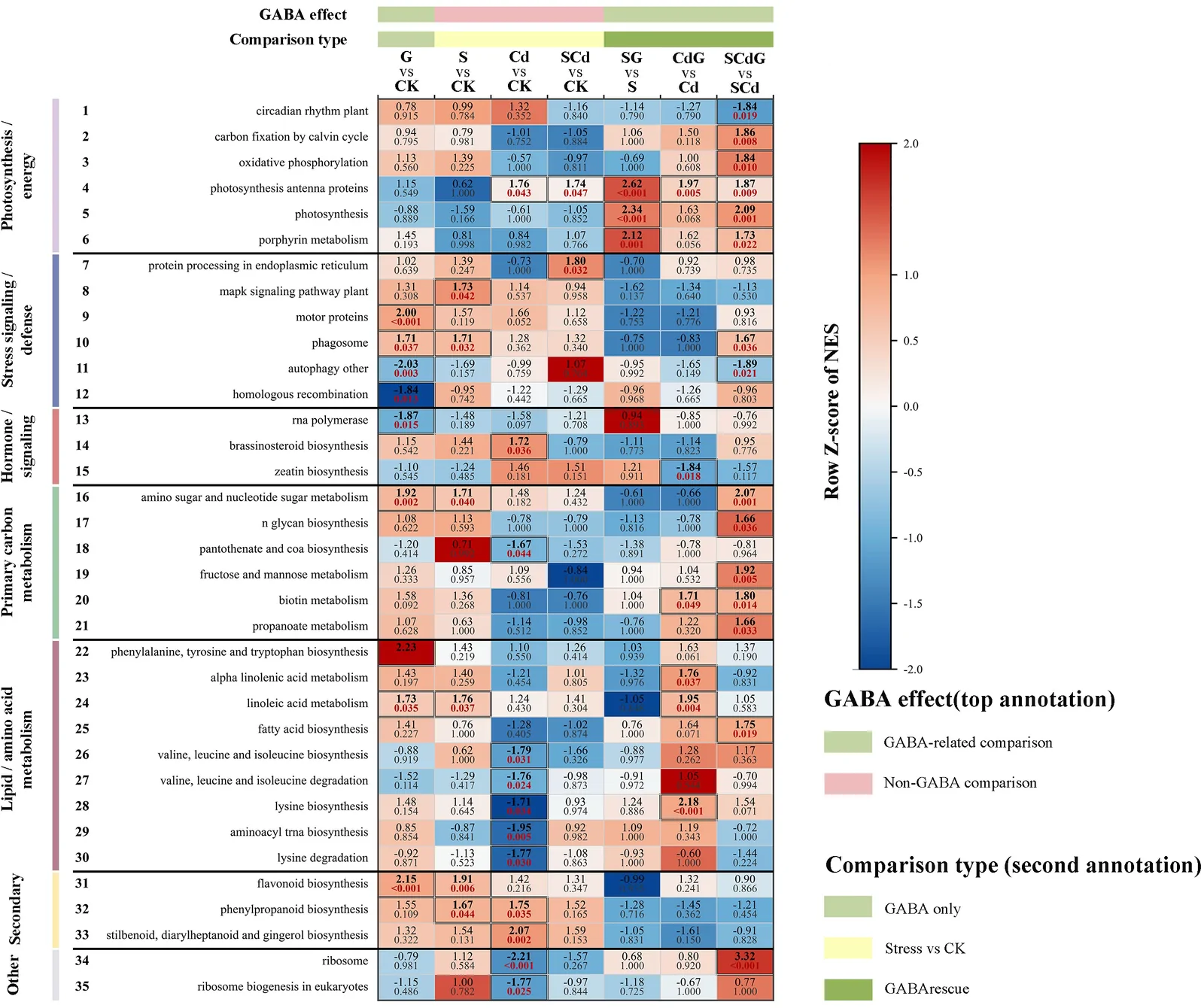
KEGG-GSEA showing significantly enriched pathways across different comparison groups. The figure displays GSEA results of significantly enriched KEGG pathways in seven comparison groups. Each row represents a KEGG pathway, and each column represents a comparison group, including G vs. CK, S vs. CK, Cd vs. CK, SCd vs. CK, SG vs. S, CdG vs. Cd, and SCdG vs. SCd. The heatmap was generated based on row-normalized normalized enrichment score (NES) values; red indicates higher relative NES values, while blue indicates lower relative NES values. Each cell displays the NES value in the upper part and the corresponding FDR q-value in the lower part. Pathways shown are those with at least one significant enrichment (FDR q-value < 0.05). Black borders indicate statistically significant enrichment in the corresponding comparison group.

KEGG-based gene set enrichment analysis (KEGG-GSEA) revealed 35 significantly enriched pathways across seven comparison groups, functionally classified into “photosynthesis” and “energy metabolism”, stress signaling and defense, “Plant hormone signal transduction”, “primary carbon metabolism”, “lipid and amino acid metabolism”, “secondary metabolism”, and other **“**metabolic process” (**Figure 14**). Overall, under stress conditions, pathways related to photosynthesis, carbon assimilation, protein synthesis, and primary metabolism generally exhibited a negative enrichment trend, indicating widespread suppression of energy capture and biosynthetic processes. In the non-GABA-treated groups, pathways related to photosynthesis, photosynthesis–antenna proteins, “Carbon fixation in photosynthetic organisms”, and “Porphyrin metabolism” consistently exhibited reduced enrichment, suggesting impairment of light energy capture and carbon assimilation capacity. In addition, stress conditions affected multiple regulatory pathways, including “MAPK signaling pathway–plant”, “Plant hormone signal transduction”, “protein processing in endoplasmic reticulum”, “Autophagy”, and “Ribosome”, indicating extensive reprogramming of signal transduction and protein homeostasis under abiotic stress. In contrast, GABA-treated groups (SG vs. S, CdG vs. Cd, and SCdG vs. SCd) showed recovery trends in these pathways, particularly in photosynthesis- and carbon metabolism-related processes, indicating that exogenous GABA contributed to the restoration of photosynthetic efficiency and carbon assimilation capacity. Furthermore, coordinated changes in “Amino sugar and nucleotide sugar metabolism”, “Fructose and mannose metabolism”, “Fatty acid biosynthesis”, “Lysine degradation”, and “Ribosome biogenesis in eukaryotes” suggest that GABA not only restored photosynthetic activity but also promoted the reprogramming of carbohydrate, lipid, amino acid, and protein metabolic networks. Collectively, these results indicate that GABA mediates system-level metabolic reprogramming involving photosynthesis, carbon metabolism, protein synthesis, and primary metabolic pathways, thereby reshaping energy allocation and promoting stress recovery in *Prunus mongolica*.

## 4 Discussion

This study showed that S, Cd, and SCd significantly inhibited seed germination, seedling establishment, and photosynthetic physiology by inducing osmotic imbalance, ion toxicity, excessive ROS accumulation, and membrane lipid peroxidation. S, Cd, and SCd significantly suppressed seed germination and seedling growth in *Prunus mongolica*, accompanied by marked accumulation of O_2_^-^, H_2_O_2_, and MDA, with SCd causing the strongest inhibitory effect, suggesting that S and Cd may exert additive or synergistic damage during the early stages of germination and seedling establishment. This result is consistent with the findings of Pacheco-Insausti et al. (2023), who reported that the combined action of salinity and Cd aggravated plant growth inhibition, membrane lipid peroxidation, and antioxidant system responses, indicating that salinity and heavy metal pollution may generate more complex combined damage effects in plants. Jiang et al. (2025) also pointed out in their study on the interaction between ROS and phytohormones that salt stress not only induces ion toxicity and osmotic stress but also triggers excessive accumulation of ROS such as O_2_^-^ and H_2_O_2_, thereby affecting the balance between plant growth and defense through the ROS–hormone signaling network, which provides a mechanistic explanation for stress-induced ROS accumulation and growth inhibition in this study. Following exogenous GABA treatment, seed germination capacity, seedling morphological traits, RV, RWC, and photosynthetic pigment contents under stress conditions were restored to varying degrees, while ROS accumulation and MDA levels were markedly reduced, indicating that GABA could effectively alleviate oxidative damage and growth inhibition caused by S, Cd, and SCd.

The recovery of seed germination and seedling morphology provides the most direct phenotypic evidence for the alleviating effect of GABA on stress-induced damage. Under stress conditions, RL elongation in *Prunus mongolica* was restricted and RV decreased, which further impaired water absorption and shoot growth, thereby inhibiting SH, SL, leaf development, and overall morphological establishment. Notably, the number of LRN increased under stress treatments, especially under SCd; this change does not necessarily indicate enhanced root function but is more likely to represent a compensatory root plasticity response triggered by the inhibition of primary root elongation and absorptive function. Zou et al. (2022) pointed out that salt stress can markedly alter the dynamic growth strategies of plant roots, and that plants often adapt to adverse soil environments by adjusting primary root elongation, lateral root formation, and root spatial distribution. Consistent with this view, GABA treatment in this study markedly improved RL, SH, and SL, while the number of LRN decreased compared with the corresponding stress groups but remained higher than that in CK, suggesting that GABA may alleviate stress-induced root architecture imbalance while maintaining a certain degree of compensatory lateral root formation, and promote the recovery of seedling morphological establishment by sustaining root metabolic activity, protecting membrane system stability, and improving water absorption capacity. However, this result is not fully consistent with the finding of Xie et al. (2019) that “Cd stress inhibits lateral root formation”. A possible explanation is that the stress system in this study involved SCd, and *Prunus mongolica*, as a desert shrub, may be more inclined to expand the local absorption interface by increasing lateral root formation after primary root inhibition; in addition, differences in treatment concentration, stress duration, and species-specific root developmental strategies may also contribute to the divergent lateral root responses.

At the physiological and biochemical levels, the central role of GABA in alleviating stress-induced damage is closely associated with the reconstruction of redox homeostasis and the enhancement of osmotic regulation. Stress-induced excessive ROS accumulation and elevated MDA levels can disrupt membrane integrity, thereby affecting root absorption, water retention, and photosynthetic system stability. Following exogenous GABA treatment, the contents of O_2_^-^, H_2_O_2_, and MDA decreased, while the activities of antioxidant enzymes, including SOD, POD, CAT, APX, and GR, increased, and the accumulation of non-enzymatic antioxidants such as AsA and GSH was enhanced, indicating that GABA may improve ROS scavenging capacity and reduce membrane lipid peroxidation damage by coordinately activating the enzymatic antioxidant system and the AsA-GSH cycle. Similarly, Li D. et al. (2022) found under S that exogenous GABA increased Chl and SS contents, reduced MDA, O_2_^-^, and H_2_O_2_ levels, and enhanced SOD, CAT, and POD activities, which is consistent with the results of this study showing that GABA alleviated oxidative damage under S and SCd. Hasanuzzaman et al. (2019) further pointed out that the AsA-GSH cycle is an important defense pathway by which plants scavenge ROS, maintain redox homeostasis, and alleviate abiotic stress-induced damage, providing theoretical support for the coordinated responses of APX, GR, AsA, and GSH observed in this study. In addition, GABA promoted the accumulation of osmotic regulatory substances, including Pro, SS, and SP, which may help maintain cellular osmotic balance, stabilize proteins and membrane structures, and improve plant water status. The recovery of photosynthetic pigments and RWC further indicates that the alleviation of oxidative damage and water imbalance by GABA can be translated into improved photosynthetic system stability and growth performance; this interpretation is also supported by Xiang et al. (2016), whose results showed that exogenous GABA alleviated S-induced damage to PSII structure and function and improved photosynthetic electron transport and energy allocation. Furthermore, Fu (2024) also found also found that GABA increased AsA and GSH contents and their redox status, and improved Chl, Car, and gas exchange parameters, indicating that the coordinated protective effect of GABA on the antioxidant system and photosynthetic system is also important under Cd. It should be noted that the regulatory effect of GABA under Cd does not consistently promote stress tolerance across all plant species and treatment methods. Song et al. (2026) found that irrigation with 0.5 mM GABA instead decreased seedling biomass and promoted Cd accumulation, which is not fully consistent with the results of this study showing that GABA alleviated Cd- and SCd-induced damage. This discrepancy may be related to plant species, GABA application concentration and method, Cd concentration, treatment stage, and root Cd uptake and transport characteristics, and also suggests that the role of GABA under heavy metal stress exhibits clear dose effects and species dependence.

Transcriptome results further revealed the molecular regulatory basis underlying the above physiological responses. DEGs analysis showed that S, Cd, and SCd all induced obvious transcriptional reprogramming in *Prunus mongolica*, among which SCd induced higher numbers of DEGs and enriched pathways, indicating that combined stress caused broader molecular perturbations to cellular functions, which was consistent with its stronger ROS accumulation, membrane lipid peroxidation, decreased RV, and water status imbalance at the physiological level. Wang et al. (2026) found that salt, Cd, and their combined stress significantly inhibited seed germination and seedling growth, induced ROS accumulation and membrane lipid peroxidation, and were accompanied by the reprogramming of pathways such as “Carbon fixation in photosynthetic organisms”, “Glyoxylate and dicarboxylate metabolism”, and “Arginine and proline metabolism”, which highly agrees with the stronger physiological damage and transcriptional perturbation induced by SCd treatment in this study. Following GABA treatment, the number of DEGs, the proportion of shared genes, and the types of enriched pathways under different stress backgrounds were not completely consistent, indicating that the regulatory effect of GABA was not a simple uniform response, but showed obvious stress type dependence. Hao et al. (2024) found that exogenous GABA alleviated Cd toxicity by improving photosynthetic characteristics, enhancing antioxidant enzyme activities, and reducing Cd uptake and accumulation, which mutually confirms the results of this study showing that GABA regulated antioxidant defense, photosynthetic pigments, and root function. *WRKY*, *bHLH*, and *MYB* transcription factor families accounted for relatively high proportions in the responses of different treatments, and these transcription factors are usually closely associated with ROS signaling, defense responses, hormone signaling, secondary metabolism, and stress adaptation, suggesting that GABA may connect external stress signals with downstream functional gene expression by reshaping transcriptional regulatory networks, thereby achieving differential regulation of defense response and growth recovery.

GO and KEGG enrichment results jointly indicated that “plasma membrane” perception, ATP-dependent processes, “defense response”, “MAPK signaling pathway–plant”, “Plant hormone signal transduction”, and peroxisome-related processes played important roles in the stress responses of *Prunus mongolica*. Changes in plasma membrane-related functions may affect ion transport, signal perception, and the maintenance of cellular homeostasis; the enrichment of ATP binding-related functions suggests that stress responses require substantial energy input, including transmembrane transport, protein kinase activation, metabolic repair, and the initiation of defense response. Further analysis showed that the MAPK cascade may participate in early stress responses by integrating Ca^2+^ signals, ROS signals, and hormone signals; the “Plant hormone signal transduction” network may regulate the balance between growth inhibition and “defense response; and peroxisome-related genes are closely associated with fatty acid metabolism”, membrane lipid peroxidation, and ROS scavenging, with their expression changes corresponding to MDA accumulation and membrane damage. Rodríguez-Serrano et al. (2009) found that Cd-induced oxidative stress significantly affected peroxisome dynamics, suggesting that peroxisomes may participate in cellular protective responses under Cd; Su et al. (2019) also pointed out that plant peroxisomes not only participate in fatty acid β-oxidation, photorespiration, and hormone biosynthesis, but also contribute to the maintenance of redox homeostasis under environmental stress through ROS/RNS metabolism. Therefore, the enrichment of peroxisome-related pathways in this study further supports the view that GABA alleviates stress-induced damage by maintaining redox balance and membrane stability.

KEGG-GSEA results showed that photosynthesis, carbon assimilation, lipid metabolism, amino acid metabolism, and ribosome-related processes were generally inhibited under stress conditions, reflecting significant constraints on energy acquisition and biosynthetic systems. Following GABA treatment, these functional modules showed certain recovery trends under different stress backgrounds, especially changes in pathways related to photosynthesis, “Carbon fixation”, sugar metabolism, lipid metabolism, and protein synthesis, indicating that GABA not only participates in the regulation of ROS homeostasis and osmotic balance, but may also improve energy allocation and support growth recovery by promoting the reconstruction of basic metabolic networks. Geng et al. (2025) found that exogenous GABA improved plant growth, photosynthetic pigments, and antioxidant metabolism, and regulated GABA shunt-related “metabolic process”, which mutually confirms the recovery trends of “photosynthesis”, “amino acid metabolism”, and basic metabolism revealed by GSEA in this study. Overall, the role of GABA in *Prunus mongolica* can be summarized as a hierarchical and progressive regulatory system: first, alleviating primary cellular damage by regulating ROS homeostasis and osmotic balance; second, coordinating defense response through “MAPK signaling pathway–plant”, “Plant hormone signal transduction”, and TF networks; and finally, achieving a dynamic balance between growth and defense by promoting the overall reprogramming of photosynthesis, carbon metabolism, and basic metabolic networks, thereby enhancing the adaptability of *Prunus mongolica* to S, Cd, and SCd.

Although the present study revealed that exogenous GABA alleviated alkali salt-, Cd-, and combined stress-induced damage through physiological regulation and transcriptomic reprogramming, genes directly associated with metal ion transport and Na^+^/K^+^ homeostasis were not systematically analyzed in this study. In addition, RNA-seq was performed using leaf tissues, whereas Na^+^, K^+^, and Cd^2+^ uptake primarily occurs in roots. Because root ion concentrations, Cd accumulation, and root-specific transcriptional responses were not directly measured, the possible roles of metal ion transporters and Na^+^/K^+^ transporters in GABA-mediated stress tolerance could not be fully determined. Future studies integrating root ionomics, Cd accumulation analysis, and root-specific transcriptome profiling will be required to clarify ion uptake and transport mechanisms under alkali salt, Cd, and combined stress conditions.

## 5 Conclusion

Our study, for the first time, systematically elucidated the multi-level regulatory mechanisms by which exogenous GABA alleviates damage induced by S, Cd, and SCd in *Prunus mongolica* at both physiological–biochemical and transcriptomic levels. The results demonstrated that combined stress induced severe inhibition of seed germination and seedling growth by triggering excessive ROS accumulation, enhanced membrane lipid peroxidation, and disruption of the photosynthetic system, with SCd exhibiting the strongest systemic damaging effects. Exogenous GABA significantly alleviated stress-induced damage by coordinating redox homeostasis and osmotic regulation, thereby re-establishing cellular metabolic balance and remodeling stress response networks. Its core regulatory effects were not only reflected in the activation of antioxidant enzyme systems and accumulation of osmoprotectants but also involved modulation of “MAPK signaling pathway–plant”, “Plant hormone signal transduction”, and peroxisome-related processes, thereby coordinating stress signal perception and defense response. Furthermore, KEGG-GSEA results revealed that GABA-mediated regulation involves system-level metabolic reprogramming, including photosynthesis, carbon metabolism, and primary metabolic networks, suggesting that GABA promotes stress recovery by optimizing cellular energy allocation strategies.

Overall, exogenous GABA alleviates S, Cd, and SCd-induced damage in *Prunus mongolica* through a hierarchical regulatory framework consisting of signal regulation, redox homeostasis maintenance, and metabolic network reprogramming. This study provides a theoretical basis for understanding stress tolerance mechanisms in xerophytic shrubs in arid regions and offers potential physiological and molecular insights for vegetation restoration in degraded ecosystems.

## Data Availability

The datasets presented in this study can be found in the National Center for Biotechnology Information (NCBI) Gene Expression Omnibus (GEO) repository under accession number GSE337475. The data are available at: https://www.ncbi.nlm.nih.gov/geo/query/acc.cgi?acc=GSE337475.
