# Supplementary material for: Exogenous γ-aminobutyric acid mitigates physiological and transcriptomic responses of Prunus mongolica under combined alkali salt and cadmium stress
Source: Front Plant Sci. 2026 Aug 25;17:1927917. doi: 10.3389/fpls.2026.1927917 (PMC13550137; doi:10.3389/fpls.2026.1927917)
Supplement: Supplementary file 1 [file SupplementaryFile1.docx]

Supplementary Material

# 1 Supplementary Figures

Supplementary Figure 1 GO functional enrichment analysis of DEGs

**Supplementary Figure 1.** (A) is the GO functional enrichment analysis of DEGs in the Cd vs. CK group; (B) is the GO functional enrichment analysis of DEGs in the G vs. CK group; (C) is the GO functional enrichment analysis of DEGs in the CdG vs. Cd group. Red represents cellular component; orange represents molecular function; blue represents biological process; green represents the number of upregulated DEGs in each GO term; yellow represents the number of downregulated DEGs in each GO term; GO IDs correspond to the unique identifiers of each functional term.

 Supplementary Figure 2 KEGG functional enrichment analysis of DEGs

**Supplementary Figure 2.** (A) is the KEGG functional enrichment analysis of DEGs in the Cd vs. CK group; (B) is the KEGG functional enrichment analysis of DEGs in the G vs. CK group; (C) is the KEGG functional enrichment analysis of DEGs in the CdG vs. Cd group. Different colors on the left represent different KEGG pathways and corresponding DEGs, while the right panel shows the KEGG pathway enrichment results of DEGs.

# 2 Supplementary Tables

Supplementary Table 1 Physicochemical properties of different soil types

| Soil type | PH | Salt content (%) | Water content (%) | Bulk density(mg/m^3^) | Porosity (%) |
| --- | --- | --- | --- | --- | --- |
| Gray desert soil | 7.57±0.03d | 4.25±0.11c | 2.16±0.39d | 6.45±1.07e | 0.23±0.04c |
| Brown calcic soil | 8.40±0.02ab | 3.52±0.25d | 8.70±1.38b | 15.65±2.43bc | 0.61±0.11ab |
| Chestnut soil | 8.56±0.15a | 1.76±0.05e | 11.57±0.82a | 23.28±0.74a | 0.78±0.08a |
| Aeolian sandy soil | 8.35±0.01bc | 6.88±0.12a | 2.66±0.39d | 7.24±1.31e | 0.26±0.05c |
| Meadow soil | 7.97±0.02c | 4.71±0.01b | 11.10±0.86a | 17.84±1.30b | 0.65±0.05ab |
| Calcareous soil | 8.33±0.04abc | 1.31±0.02f | 2.53±0.79d | 1.27±0.20f | 0.05±0.01c |
| Coal cinder soil | 8.14±0.05bc | 4.72±0.01b | 6.11±0.92c | 10.18±2.09d | 0.61±0.32ab |
| Clay soil | 7.31±0.56d | 1.72±0.03e | 6.24±0.32c | 14.44±0.77c | 0.50±0.04b |

**Supplementary Table 1.** Values are expressed as mean ± standard deviation (n = 3). Different lowercase letters within a column indicate statistically significant differences among treatments (p < 0.05), determined by one-way ANOVA followed by Tukey’s HSD test.

Supplementary Table 2 Physicochemical properties of the experimental substrate

| Substrate | Particle size（mm） | PH | EC（mS/cm） | Nutrient elements（kg/m^3^） | N (mg/L) | Phosphate (mg/L) | K (mg/L) |
| --- | --- | --- | --- | --- | --- | --- | --- |
| Fertilized white peat substrate | 0.00-5.00 | 5.00-6.00（CaCl_2_） | ＜1.00 | 0.80 | 110.00 | 130.00 | 140.00 |

**Supplementary Table 2.** The values represent the physicochemical specifications of the experimental substrate provided by the manufacturer. pH was determined using the CaCl₂ method; EC indicates electrical conductivity; N, phosphate, and K represent the available mineral nutrient contents.

Supplementary Table 3 Primers used for qRT-PCR validation of RNA-seq identified genes

| Gene ID | Forward primer (5′–3′) | Reverse primer (5′–3′) |
| --- | --- | --- |
| β-actin | ACTGGAATGGTTAAGGCTGG | CATAGGCATCTTTCTGTCCC |
| Pmo01G023290 | CCAGTCCATAAGAAAGTCCC | GTCGGAAGAGAGGATAGATG |
| Pmo04G010260 | ATCGAGGAAGAGAGAAGTGC | GTTGTTGCACGAAATCCCTC |
| Pmo08G004790 | CTCTTGGTGTCATGCATAGG | CTTGTCCTGGCTTGTAGAAG |
| Pmo08G020230 | GCAGAGGCATTAACAGAAGC | GTCCAGTGATTGGATCACTG |
| Pmo02G026290 | CTGTGACCCAAGTACCAATG | GAAGTGAGAGAGTAGCTTCC |

**Supplementary Table 3.** β-actin was used as the reference gene for normalization of qRT-PCR data. Primer sequences were used for the validation of RNA-seq identified differentially expressed genes (DEGs). All primer sequences are presented in the 5′–3′ direction.

Supplementary Table 4 Quality assessment results of RNA extraction

| Sample | concentration (µg/µL) | RIN | Amount (µg) |
| --- | --- | --- | --- |
| CK_1 | 0.348 | 7.900 | 17.387 |
| CK_2 | 0.276 | 7.200 | 13.811 |
| CK_3 | 0.306 | 8.200 | 15.300 |
| G_1 | 0.432 | 7.400 | 21.625 |
| G_2 | 0.367 | 8.400 | 18.358 |
| G_3 | 0.255 | 8.100 | 12.743 |
| S_1 | 0.368 | 8.200 | 18.381 |
| S_2 | 0.202 | 8.100 | 10.114 |
| S_3 | 0.270 | 8.100 | 13.504 |
| Cd_1 | 0.385 | 7.400 | 19.251 |
| Cd_2 | 0.384 | 8.300 | 19.200 |
| Cd_3 | 0.314 | 7.800 | 15.714 |
| SCd_1 | 0.332 | 7.900 | 16.612 |
| SCd_2 | 0.350 | 7.900 | 17.519 |
| SCd_3 | 0.363 | 8.000 | 18.171 |
| SG_1 | 0.242 | 8.100 | 12.098 |
| SG_2 | 0.361 | 8.400 | 18.059 |
| SG_3 | 0.185 | 8.200 | 9.272 |
| CdG_1 | 0.159 | 7.900 | 7.958 |
| CdG_2 | 0.270 | 8.200 | 13.491 |
| CdG_3 | 0.187 | 7.300 | 9.353 |
| SCdG_1 | 0.187 | 7.300 | 9.353 |
| SCdG_2 | 0.377 | 8.400 | 18.833 |
| SCdG_3 | 0.321 | 8.000 | 16.025 |

**Supplementary Table 4.** RIN represents the RNA integrity number, which was used to evaluate RNA integrity. The suffixes _1, _2, and _3 indicate biological replicates. CK, G, S, Cd, SCd, SG, CdG, and SCdG represent the control, GABA treatment, alkali-salt stress, Cd stress, combined alkali-salt and Cd stress, alkali-salt stress plus GABA, Cd stress plus GABA, and combined alkali-salt and Cd stress plus GABA treatments, respectively.

Supplementary Table 5 Summary of RNA-seq data quality for different treatment groups of *Prunus mongolica*.

| Sample | Raw Reads | Clean Reads | Clean reads Ratio(%) | Q (20%) | Q (30%) | GC content(%) |
| --- | --- | --- | --- | --- | --- | --- |
| CK_1 | 43891050 | 42254148 | 96.27 | 100 | 99.28 | 45.50 |
| CK_2 | 34380014 | 33027782 | 96.07 | 100 | 99.27 | 45.50 |
| CK_3 | 41613306 | 39988114 | 96.09 | 100 | 99.3 | 45.50 |
| G_1 | 42243586 | 40524660 | 95.93 | 100 | 99.27 | 45.50 |
| G_2 | 40963750 | 39478670 | 96.37 | 100 | 99.28 | 45.50 |
| G_3 | 43867086 | 41898952 | 95.51 | 100 | 99.26 | 45.50 |
| S_1 | 41621836 | 40153150 | 96.47 | 100 | 99.28 | 45.50 |
| S_2 | 41649260 | 40042760 | 96.14 | 100 | 99.28 | 45.50 |
| S_3 | 41863944 | 40065244 | 95.7 | 100 | 99.28 | 45.50 |
| Cd_1 | 42453042 | 40801986 | 96.11 | 100 | 99.28 | 45.50 |
| Cd_2 | 34332336 | 32819298 | 95.59 | 100 | 99.27 | 46.00 |
| Cd_3 | 43523980 | 41690764 | 95.79 | 100 | 99.29 | 45.50 |
| SCd_1 | 40974354 | 39346988 | 96.03 | 100 | 99.25 | 45.50 |
| SCd_2 | 41047684 | 39493748 | 96.21 | 100 | 99.27 | 45.50 |
| SCd_3 | 42201134 | 40790510 | 96.66 | 100 | 99.26 | 46.00 |
| SG_1 | 38944436 | 37510548 | 96.32 | 100 | 99.28 | 45.50 |
| SG_2 | 41470608 | 39807830 | 95.99 | 100 | 99.27 | 46.00 |
| SG_3 | 34099194 | 32553344 | 95.47 | 100 | 99.25 | 46.00 |
| CdG_1 | 42177962 | 40597900 | 96.25 | 100 | 99.28 | 46.00 |
| CdG_2 | 41224728 | 39584656 | 96.02 | 100 | 99.29 | 45.50 |
| CdG_3 | 41688906 | 40286438 | 96.64 | 100 | 99.28 | 45.50 |
| SCdG_1 | 42409352 | 40836866 | 96.29 | 100 | 99.28 | 46.00 |
| SCdG_2 | 35357636 | 34283032 | 96.96 | 100 | 99.28 | 45.50 |
| SCdG_3 | 42378230 | 41043460 | 96.85 | 100 | 99.26 | 46.00 |

**Supplementary Table 5.** Raw reads indicate the total number of reads obtained from the raw sequencing data. Clean reads indicate the number of high-quality reads retained after quality filtering. Clean reads ratio represents the proportion of clean reads relative to raw reads. Q20 and Q30 represent the percentages of bases with Phred quality scores greater than 20 and 30, corresponding to sequencing error rates lower than 1% and 0.1%, respectively. GC content indicates the proportion of guanine and cytosine bases in the clean reads.

Supplementary Table 6 GO classification statistics of DEGs in different comparison groups.

| Comparison | DEGs | Total GO terms | Category | GO Terms |
| --- | --- | --- | --- | --- |
| S vs. CK | 655 | 344 | Biological Process | 164 |
|  |  |  | Cellular Component | 38 |
|  |  |  | Molecular Function | 142 |
| Cd vs. CK | 271 | 169 | Biological Process | 79 |
|  |  |  | Cellular Component | 27 |
|  |  |  | Molecular Function | 63 |
| SCd vs. CK | 1425 | 643 | Biological Process | 361 |
|  |  |  | Cellular Component | 71 |
|  |  |  | Molecular Function | 211 |
| G vs. CK | 655 | 389 | Biological Process | 198 |
|  |  |  | Cellular Component | 49 |
|  |  |  | Molecular Function | 142 |
| SG vs. S | 1341 | 586 | Biological Process | 323 |
|  |  |  | Cellular Component | 71 |
|  |  |  | Molecular Function | 192 |
| CdG vs. Cd | 505 | 293 | Biological Process | 146 |
|  |  |  | Cellular Component | 29 |
|  |  |  | Molecular Function | 118 |
| SCdG vs. SCd | 323 | 228 | Biological Process | 111 |
|  |  |  | Cellular Component | 31 |
|  |  |  | Molecular Function | 86 |

**Supplementary Table 6.** DEGs indicate differentially expressed genes. GO terms were classified into three main categories: biological process, cellular component, and molecular function. The column “Total GO terms” represents the total number of GO terms annotated in each comparison, while “GO terms in each category” represents the number of GO terms assigned to each GO category.

Supplementary Table 7 KEGG pathway classification of differentially expressed genes in different comparison groups.

| Comparison | KEGG-annotated DEGs | Total KEGG pathways | KEGG pathway category | Pathways in each category |
| --- | --- | --- | --- | --- |
| S vs. CK | 76 | 44 | Cellular Processes | 3 |
|  |  |  | Environmental Information Processing | 3 |
|  |  |  | Genetic Information Processing | 2 |
|  |  |  | Metabolism | 35 |
|  |  |  | Organismal Systems | 1 |
| Cd vs. CK | 11 | 11 | Cellular Processes | 1 |
|  |  |  | Environmental Information Processing | 2 |
|  |  |  | Metabolism | 7 |
|  |  |  | Organismal Systems | 1 |
| SCd vs. CK | 110 | 49 | Cellular Processes | 4 |
|  |  |  | Environmental Information Processing | 3 |
|  |  |  | Genetic Information Processing | 6 |
|  |  |  | Metabolism | 34 |
|  |  |  | Organismal Systems | 2 |
| G vs. CK | 62 | 40 | Cellular Processes | 1 |
|  |  |  | Environmental Information Processing | 3 |
|  |  |  | Genetic Information Processing | 4 |
|  |  |  | Metabolism | 30 |
|  |  |  | Organismal Systems | 2 |
| SG vs. S | 61 | 32 | Cellular Processes | 2 |
|  |  |  | Environmental Information Processing | 2 |
|  |  |  | Genetic Information Processing | 3 |
|  |  |  | Metabolism | 23 |
|  |  |  | Organismal Systems | 2 |
| CdG vs. Cd | 36 | 24 | Environmental Information Processing | 2 |
|  |  |  | Genetic Information Processing | 2 |
|  |  |  | Metabolism | 19 |
|  |  |  | Organismal Systems | 1 |
| SCdG vs. SCd | 17 | 16 | Cellular Processes | 1 |
|  |  |  | Genetic Information Processing | 5 |
|  |  |  | Metabolism | 8 |
|  |  |  | Organismal Systems | 2 |

**Supplementary Table 7.** DEGs indicate differentially expressed genes. “KEGG-annotated DEGs” represents the number of DEGs assigned to KEGG pathways in each comparison. “Total KEGG pathways” represents the total number of KEGG pathways annotated in each comparison, while “Pathways in each category” represents the number of pathways assigned to each KEGG pathway category. Differences in the numbers between GO and KEGG annotations are due to differences in database coverage, annotation criteria, and functional classification systems.
